# A systematic review and meta-analysis of heart rate variability in COPD

**DOI:** 10.3389/fcvm.2023.1070327

**Published:** 2023-02-17

**Authors:** Jaber S. Alqahtani, Abdulelah M. Aldhahir, Saeed M. Alghamdi, Shouq S. Al Ghamdi, Ibrahim A. AlDraiwiesh, Abdullah S. Alsulayyim, Abdullah S. Alqahtani, Nowaf Y. Alobaidi, Lamia Al Saikhan, Saad M. AlRabeeah, Eidan M. Alzahrani, Alessandro D. Heubel, Renata G. Mendes, Abdullah A. Alqarni, Abdullah M. Alanazi, Tope Oyelade

**Affiliations:** ^1^Department of Respiratory Care, Prince Sultan Military College of Health Sciences, Dammam, Saudi Arabia; ^2^Respiratory Therapy Department, Faculty of Applied Medical Sciences, Jazan University, Jazan, Saudi Arabia; ^3^Respiratory Care Program, Clinical Technology Department, College of Applied Health Science, Umm Al Qura University, Makkah, Saudi Arabia; ^4^Anesthesia Technology Department, Prince Sultan Military College of Health Sciences, Dammam, Saudi Arabia; ^5^National Heart and Lung Institute, Imperial College London, London, United Kingdom; ^6^Respiratory Therapy Department, King Saud bin Abdulaziz University for Health Sciences, Alahsa, Saudi Arabia; ^7^Department of Cardiac Technology, College of Applied Medial Sciences, Imam Abdulrahman Bin Faisal University, Dammam, Saudi Arabia; ^8^Physical Therapy Department, Prince Sultan Military College of Health Sciences, Dammam, Saudi Arabia; ^9^Cardiopulmonary Physiotherapy Laboratory, Department of Physical Therapy, Federal University of São Carlos, SP, Brazil; ^10^Department of Respiratory Therapy, Faculty of Medical Rehabilitation Sciences, King Abdulaziz University, Jeddah, Saudi Arabia; ^11^Department of Respiratory Therapy, College of Applied Medical Sciences, King Saud bin Abdulaziz University for Health Sciences, Riyadh, Saudi Arabia; ^12^King Abdullah International Medical Research Center, Riyadh, Saudi Arabia; ^13^UCL Institute for Liver and Digestive Health, London, United Kingdom

**Keywords:** heart rate variability, COPD, sympatho-vagal, ECG, prognosis

## Abstract

**Background:**

Chronic obstructive pulmonary disease (COPD) is associated with disruption in autonomic nervous control of the heart rhythm. We present here quantitative evidence of the reduction in HRV measures as well as the challenges to clinical application of HRV in COPD clinics.

**Method:**

Following the Preferred Reporting Items for Systematic Reviews and Meta-Analyses (PRISMA) guidelines, we search in June 2022 Medline and Embase databases for studies reporting HRV in COPD patients using relevant medical subject headings (MeSH) terms. The quality of included studies was assessed using the modified version of the Newcastle–Ottawa Scale (NOS). Descriptive data were extracted, while standardized mean difference was computed for changes in HRV due to COPD. Leave-one-out sensitivity test was performed to assess exaggerated effect size and funnel plots to assess publication bias.

**Results:**

The databases search yielded 512 studies, of which we included 27 that met the inclusion criteria. The majority of the studies (73%) had a low risk of bias and included a total of 839 COPD patients. Although there were high between-studies heterogeneity, HRV time and frequency domains were significantly reduced in COPD patients compared with controls. Sensitivity test showed no exaggerated effect sizes and the funnel plot showed general low publication bias.

**Conclusion:**

COPD is associated with autonomic nervous dysfunction as measured by HRV. Both sympathetic and parasympathetic cardiac modulation were decreased, but there is still a predominance of sympathetic activity. There is high variability in the HRV measurement methodology, which affects clinical applicability.

## Introduction

Chronic obstructive pulmonary disease (COPD), the third cause of mortality worldwide, remains to be a major public health problem (1). COPD is a respiratory condition/disease characterized by chronic obstruction of the airflow with pathological changes in the lung clustered with important comorbidities/risk factors and significant systemic/extrapulmonary effects all of which may contribute to the severity of the disease (2, 3). COPD, as a chronic illness, may influence the autonomic nervous system (ANS) function, introducing pathophysiological alternations to the sympathetic vagal balance (4). This could include, but not limited to sympathetic overestimation and/or a reduction in vagal activity (4).

Alterations in ANS function can be assessed non-invasively using heart rate variability (HRV), a measure of cardiac autonomic function (5). HRV, defined as fluctuations in both RR intervals and instantaneous heart rates between consecutive heartbeats (5, 6), is directly linked to the behavior/function of sinus node and is considered a biomarker of health (6). Higher HRV indicates a greater adaptation by the cardiovascular system for intrinsic and extrinsic changes such as exercise or stress, whereas lower HRV indicates a greater risk/predisposition for cardiovascular mortality and morbidity (6, 7).

Cardiac autonomic function is adversely affected in patients with COPD. Multiple/small individual studies have reported reduced HRV in patients with COPD compared to match healthy controls, and indexes of HRV may relate to disease severity (8–11). A previous systematic review, conducted in 2014 and included reports published in the last 6 years from the date of the review, included only a qualitative synthesize, and did not follow PRISMA guidelines in conducting and reporting systematic review and meta-analysis (12). Yet, no previous systematic review and meta-analysis has been performed, which quantitatively synthesized the level of evidence of HRV in patients with COPD.

The aim of this study was to assess and identify how COPD affects autonomic nervous control of heart rhythm as measured by HRV and how that change is related to COPD severity.

## Methods

This systematic review and meta-analysis followed the Preferred Reporting in Systematic Reviews and Meta-Analyses (PRISMA) guidelines (13) and was registered *a priori* to Prospero (registration number is: CRD42020172268).

Two databases (MEDLINE and EMBASE) were searched from inception till July 2022 employing medical subject headings (MeSH) terms. The search strategy was designed to be extensive and cover relevant studies, according to the search algorithm of databases (Supplementary Tables S1, S2). Duplicated studies were removed using EndNote algorithm to screen for similar title, publication dates and authors. The Rayyan intelligent systematic review software was used for title, abstract and full text screening of the duplicate-free studies.

### Patient and public involvement statement

There was no involvement of patient and public in this paper.

### Inclusion criteria

Eligibility of studies was based on the use of any indices of HRV to access the non-cardiac, physiological control of heart rate in patients with COPD. Studies not using the conventional measuring techniques for HRV (e.g., tilting technique) based on the guidelines of the European Society of cardiology and the North American Society of Pacing and Electrophysiology were excluded (14). Also, studies involving pharmacological interventions previously reported to influence HRV were excluded. Thus, interventional studies/trials assessing the effect of pharmacological or non-pharmacological treatments on COPD or HRV in COPD patients were excluded.

### Studies screening

The title and abstract screening of studies was independently performed by two of the authors (JS and TO). To identify studies based on the inclusion criteria, two authors screen the full-texts articles. All conflicts were resolved *via* online meetings mediated by a third author (AMA).

### Quality assessment

The evaluation of the quality of the methods employed in each of the included studies was done by two of the authors (SM and NA) using the modified Newcastle-Ottawa Scale (NOS) (15). The modified NOS included seven domains, each of which was assessed from 0 (high risk of bias) to 3 (low risk of bias), and we averaged the domain scores to get a score between 0 and 3, where a higher number reflects a lower risk of bias. Any discrepancies in the quality rating were settled by discussion with a third author.

### Data synthesis and analysis

According to predefined criteria, we extracted the title, location, design, aims and summary findings of included studies into a predefined table. Further, we extracted the sample sizes (of COPD and healthy controls), gender, age, FEV_1_% of COPD patients, the ECG recording equipment use, length of ECG analyzed, sampling frequency of ECG, software used for HRV analysis, HRV indices computed and HRV indices that were reported to be influenced by COPD (Table 1). Meta-analysis was performed to compute the standardized mean differences (SMD) with the 95% confidence interval (CI) of the HRV indices reported. Forest plots of SMDs between HRV of COPD patients and matched controls were computed using the *Metan* algorithm in Stata/SE16. SMD was used as a measure of the effect size to correct for the heterogeneity in the studies design and analysis (duration of ECG or number of NN-interval analyzed). Random or fixed effect model was used according to the between-study heterogeneity measured by the I^2^ statistic. According to Hopkins et al., SMDs are interpreted as trivial (<0.2), small (0.2–0.6), moderate (0.6–1.2), large (1.2–2.0), very large (2.0–4.0) and extremely large (>4.0) based on the magnitude of differences observed between the groups (39). Where HRV indices were reported as median and interquartile ranges, conversion to mean ± standard deviation was performed as described by Hozo et al. (40). Sensitivity test using the “meta, leave-one-out” algorithm on Stata was performed where computed studies are more than two to test whether any of the studies within the meta-analyzed set is exaggerating the effect size. We did not perform for analysis involving two studies as it will be impossible to analyze one study if the other was excluded. Also, the definition of the various HRV indices available is presented in Supplementary Table S4.

**Table 1:** ECG recording and HRV analysis techniques of included studies.

Study	Aim	Country	Sample size (Male; COPD/Control)	Age Mean ± SD or Median (IQR; COPD/Control)	COPD GOLD severity (based on FEV1%)
Incalzi et al., 2009 (16)	To assess the relationship between autonomic dysfunction and COPD severity	Italy	55 (46)	69.1 ± 7.7	Not reported
Bartels et al. 2003 (17)	To evaluate cardiac autonomic modulation in patients with COPD during peak exercise	United States	53 (27)/14(7)	63 ± 10/60 ± 8	Not reported
Bedard et al., 2010 (18)	To compare in COPD patients and healthy controls during normal daily life and to evaluate the influence of anticholinergic andβ-adrenergic medications on HRV in COPD patients	Canada	41 (28)/19(14)	67 ± 7	Not reported
Borghi-Silva et al., 2008 (19)	To evaluate the acute effects of bi-level positive airway pressure (BiPAP) on heart rate variability (HRV) of stable chronic obstructive pulmonary disease patients (COPD).	Brazil	19 (19)/8(8)	69 ± 8	GOLD-3 (FEV1 = 35 ± 9)
Camillo et al., 2008 (8)	To study the relationship between HRV and different disease characteristics which indicate disease severity and the degree of pulmonary, muscular, and functional impairment in patients with COPD, such as the BODE index and its variables, exercise capacity, respiratory and peripheral muscle force, body composition, and level of physical activity in daily life	Brazil	31 (16)	66 ± 8	FEV1% (46 ± 15)
Carvalho et al., 2011 (20)	To evaluate short- and long-term fractal exponents of HRV in COPD subjects	Brazil	15 (10)/15(8)	73.93 ± 6.61/ 68.73 ± 7.27	FEV1% (51.69 ± 16.32)
Chang et al., 2011 (21)	To noninvasively investigate cardiac autonomic modulation in patients with severe COPD during heavy and very heavy exercise at and above the CP.	United States	9 (6)	60.2 ± 6.9	Not reported
Chen et al., 2006 (9)	To examine the relationship between the derangements in the cardiac autonomic nervous function and the oxygenation status or degree of airflow obstruction in COPD patients by using HRV analysis	Taiwan	30(25)/18(15)	69.6 ± 6.5/64.8 ± 9.0	FEV1% (50.5 ± 19.9)
Corbo et al., 2013 (22)	To assess whether HRV at rest and during physical activity, is influenced by the severity of the COPD and whether the influence is related to systemic inflammation.	Italy	30(25)	65.92 ± 9.73	FEV1% (47.72 ± 18.32)
Goulart et al., 2016 (23)	To assess if alterations in respiratory muscle strength may affect cardiac autonomic modulation in COPD patients	Brazil	10(8)	61.2 ± 6.7	FEV1% (31.9 ± 13.6)
Gunduz et al., 2009 (24)	To evaluate the HRV and HRT variables in COPD patients	Turkey	25(22)/25(19)	63 ± 7/60 ± 8	FEV1% (44 ± 15)
Surulichamy et al. 2017 (25)	To access the HRV in patients with COPD and to compare with normal individuals.	India	30(23)/30(23)	43.72 ± 4.42/43.72 ± 4.34	Not reported
Leite et al., 2015 (26)	To assess the link between resting heartrate variability (HRV) indexes with aerobic physiological variables obtained at a maximal exercise test in patients with COPD	Brazil	36(22)	63 (59–70)	FEV1%(46 (35.4–63.7))
Lu et al., 2016 (27)	To access the clinical significance of the cross-spectral measures of ECG and nostril airflow signals in COPD patients	Taiwan	23(19)/23(20)	81 (76–83)/ 77 (76–78)	FEV1%(66.9 (55.6–90.6))
Mazzuco et al., 2015 (28)	Ta access whether impairment of static lung volumes and lung diffusion capacity could be related to HRV indices in patients with moderate to severe COPD	Brazil	16 (16)	66.3 ± 8.4	FEV1%(53.9 ± 19.7)
Mendes et al., 2011 (29)	To analyse heart rate (HR), blood pressure and heart rate variability in COPD patients undergoing FVC testing	Brazil	29 (29)	72 ± 8	53.1 ± 29.2
Pan et al., 2018 (30)	To access whether indoor particulate matter and black carbon may affect the HRV/HR in patients with COPD.	China	43(40)	71.49 ± 6.40	FEV1% (< 80%)
Reis et al., 2010 (31)	To evaluate the influence of respiratory muscle strength on the magnitude of respiratory sinus arrhythmia.	Brazil	10(10)/9(9)	69 ± 9	FEV1% (41 ± 11)
Sima et al. 2017 (32)	To assess the test–retest reliability of HRV measurement from short-term ECG recording performed during spontaneous breathing in individuals with moderate-to-severe COPD.	Canada	13 (8)	63 ± 6	FEV1% (46 ± 16)
Stein et al., 1998 (10)	To determine if HRV is decreased or reflects severity in COPD and whether HRV is affected by PiZ α1-antitrypsin deficiency.	United States	13(NR)/13(NR)	42 ± 5	Not reported
Tseng et al., 2018 (33)	To noninvasively evaluate cardiac autonomic modulation in patients with COPD, during acute exacerbation	Taiwan	33(32)	77.1 ± 1.6	GOLD stage 2 (16) GOLD stage 3 (17)
Tukek et al., 2003 (34)	To assess the possible effect of diurnal variability of heart rate on the development of arrhythmias in patients with COPD	Turkey	41(39)/32(27)	59 ± 8.5/57 ± 11	FEV1% (40 ± 16)
Gestel et al., 2011 (35)	To investigate if cardiac autonomic dysfunction plays a role in HRQL in patients with COPD	Germany	60(23)	65.2 ± 7.7	FEV1% (46.58 ± 18.53)
Vanzella et al., 2018 (36)	To evaluate autonomic modulation in individuals with and without COPD	Brazil	43(NR)/31(NR)	66.37 ± 8.27/63.25 ± 7.13	FEV1% (54.79 ± 21.04)
Volterrani et al., 1994 (11)	To evaluate the presence of autonomic dysfunction in patients with COPD compared with normal population using HRV	Italy	31(31)/32(32)	55 ± 10/NR(age-matched)	FEV1% (52 ± 8.3)
Zamarron et al., 2014 (37)	To analyse heart rate variability in COPD patients under stable condition and during acute exacerbation episodes (AECOPD)	Spain	23(23)/8(8)	69.6 ± 7.3/68.6 ± 4.9	Not reported
Castello-Simões wt al. 2021 (38)	To investigate the brain-heart autonomic axis function across different clinical status and severity of COPD	Brazil	77 (50)	65.5 ± 8	Gold 1–4

## Results

**Figure 1:**
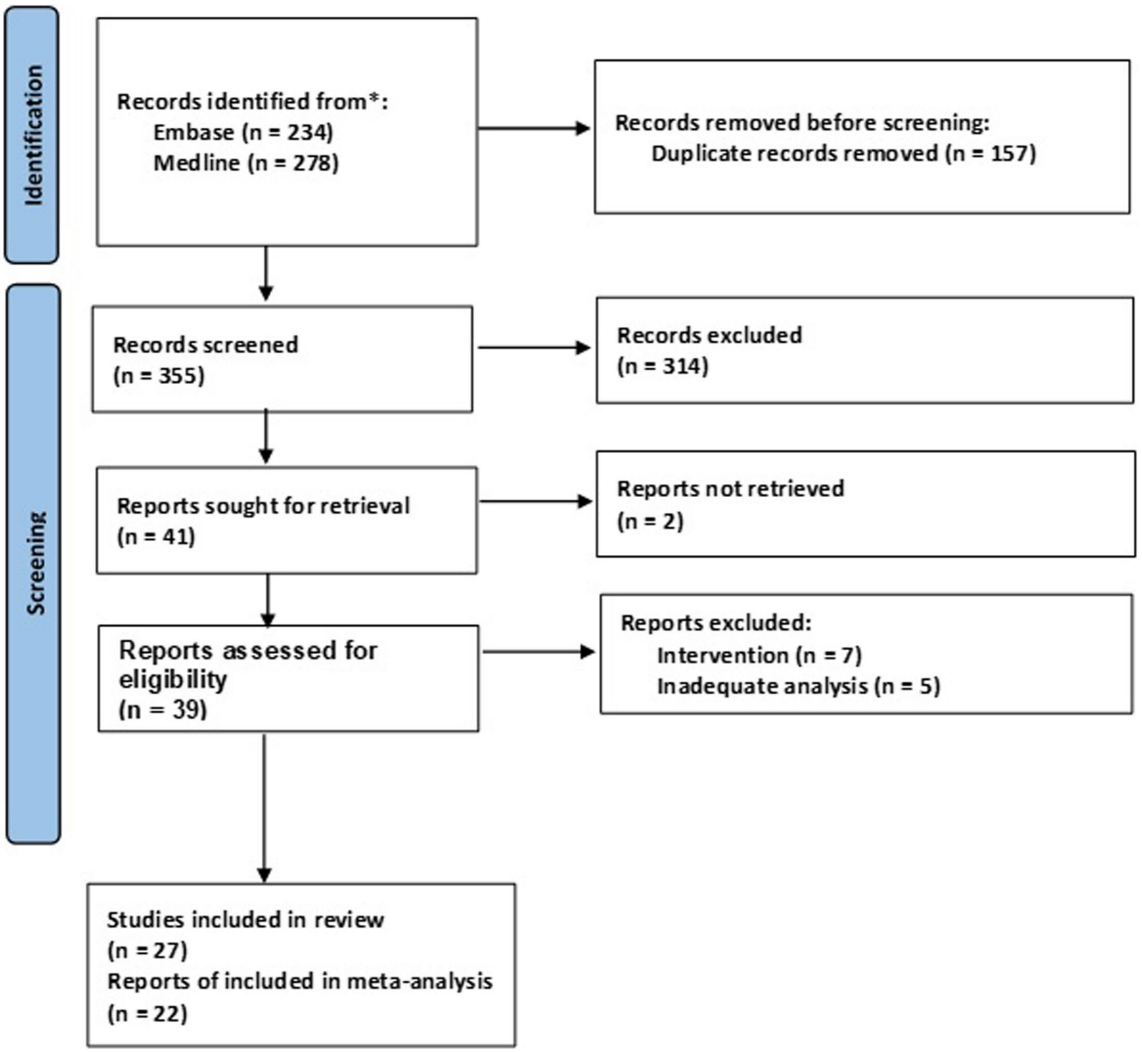
PRISMA flow chart for the included studies.

The databases search generated a total of 512 studies including 157 duplicates. Initial title and abstract screening resulted in a total of 41 studies which were potentially eligible based on the inclusion/exclusion criteria. Two of the 41 studies did not have retrievable full texts and were excluded. Full text screening of the resulting studies resulted in a further exclusion of 12 studies to give a total of 27 studies eligible for inclusion in the systematic review or meta-analysis (Figure 1).

### Features of included studies

Included studies involve a total of 839 patients clinically diagnosed as having COPD with sample size ranges between 10 and 77. Majority of the studies were case control (14/26) with 9 (33%) conducted in Brazil (Table 1). Of the 27 observational studies, 27% (7/26) had a high risk of bias, whereas the rest scored more than 1.5 in the risk assessment, indicating a low risk of bias (Supplementary Table S3). More details about the included studies presented in the Supplementary Table S1.

### Time domain HRV indices in COPD

Twenty two of the 26 included studies analyzed time domain indices of HRV in COPD patients including the standard deviation of NN intervals (SDNN), SDNN Index (SDNNI), standard deviation of the average NN intervals for each 5 min segment of a 24 h ECG recording (SDANN), root mean square of successive NN interval (RMSSD), coefficient of variation of NN intervals (i.e., SDNN/mean NN), count and percentage of NN intervals that differ by 50% (NN50 and pNN50). Of the 22 studies, 8 compared the indices in COPD patients with healthy controls and were included in the computation for effect sizes.

#### SDNN

**Figure 2:**
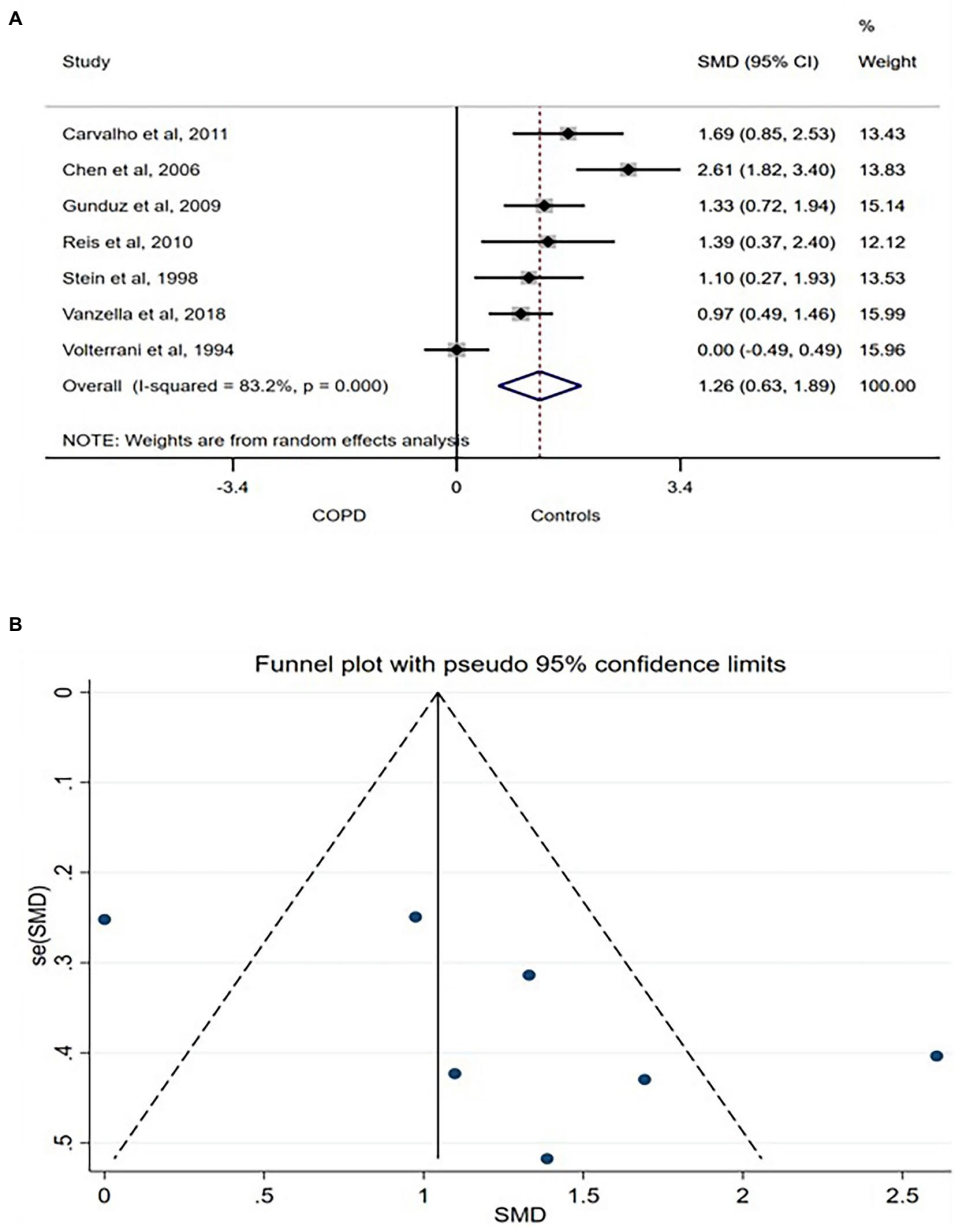
SDNN forest plot **(A)** showing the SMD and funnel plot **(B)** assessing the presence of publication bias.

SDNN related to the influence of both arms of the ANS on the heart rhythm. Thus, higher SDNN is associated with interpreted as a strong physiological connection between the ANS and the heart (6). Seven studies compared SDNN between COPD patients and age- and/or sex-matched control and were included in the meta-analysis. A large [SMD (95% CI) = 1.26 (0.063–1.89)] was found in between the groups with lower SDNN consistently reported in COPD patients (Figure 2A). The between-studies heterogeneity was significantly high. However, the funnel plot showed no publication bias (Figure 2B) while sensitivity test using the leave-one-out analysis showed no exaggerated effect (Supplementary Figure S1).

#### rMSSD

**Figure 3:**
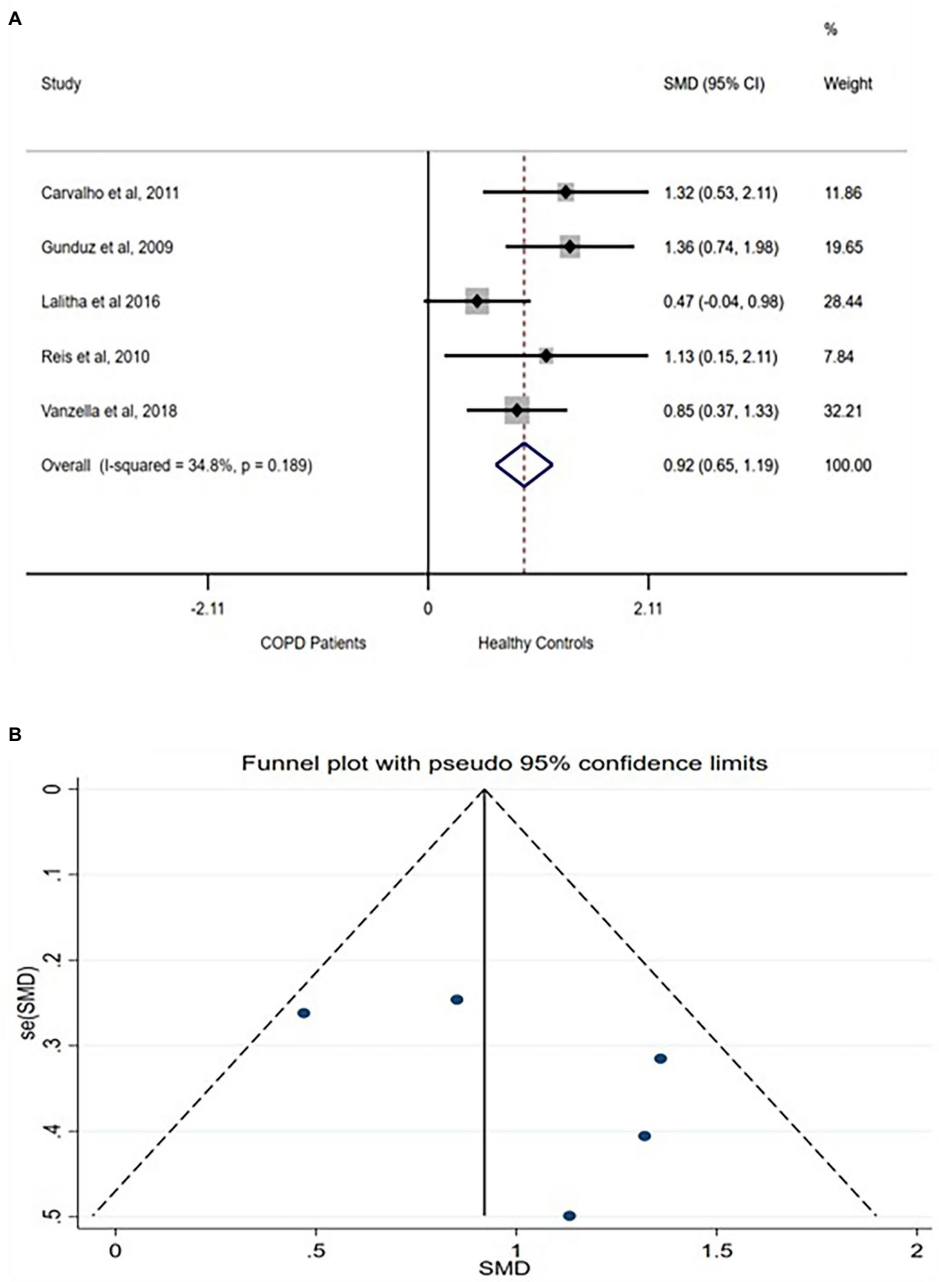
rMSSD forest plot **(A)** showing the SMD and funnel plot **(B)** assessing the presence of publication bias.

rMSSD represents the root means square of successive NN intervals and is physiologically translated as the measure of the total parasympathetic, vagally controlled variation in the heart rhythm. Five of the included studies compared the rMSSD between COPD patients and healthy controls. A moderate difference in rMSSD [SMD (95% CI = 0.92) (0.65–1.19)] was found between the group with no between-studies heterogeneity (Figure 3A). The funnel plot also showed no publication bias (Figure 3B) while sensitivity test showed no exaggerated effects (Supplementary Figure S2).

##### Other Time Domain Indices

Two studies each compared SDNNI, SDANN, NN50, and pNN50 between COPD patients and healthy controls. All studies reported significant difference with lower values reported in COPD patients compared with healthy controls (Supplementary Figures S3–S6). Full definition and physiological interpretation of these indices are reported elsewhere (6).

##### Frequency domain HRV indices in COPD

Of the 26 studies, 25 reported HRV frequency domains in COPD. Studies used frequency domain of HRV measurement in COPD patients. Eight studies compared HRV frequency domain indices between COPD patients and healthy controls and were analyzed. Indices reported include total power (TP) which represents the energy of all frequencies bands of ECG time series; high frequency power (HF); the power within the high frequency band (HF; 0.15–0.40 Hz), low frequency band (LF; 0.04–0.15 Hz), very low frequency band (VLF; 0.0033–0.04 Hz) and ultra-low frequency band (ULF; ≤0.003 Hz) as well as the ratio of LF to HF (F:HF).

#### Total power

**Figure 4:**
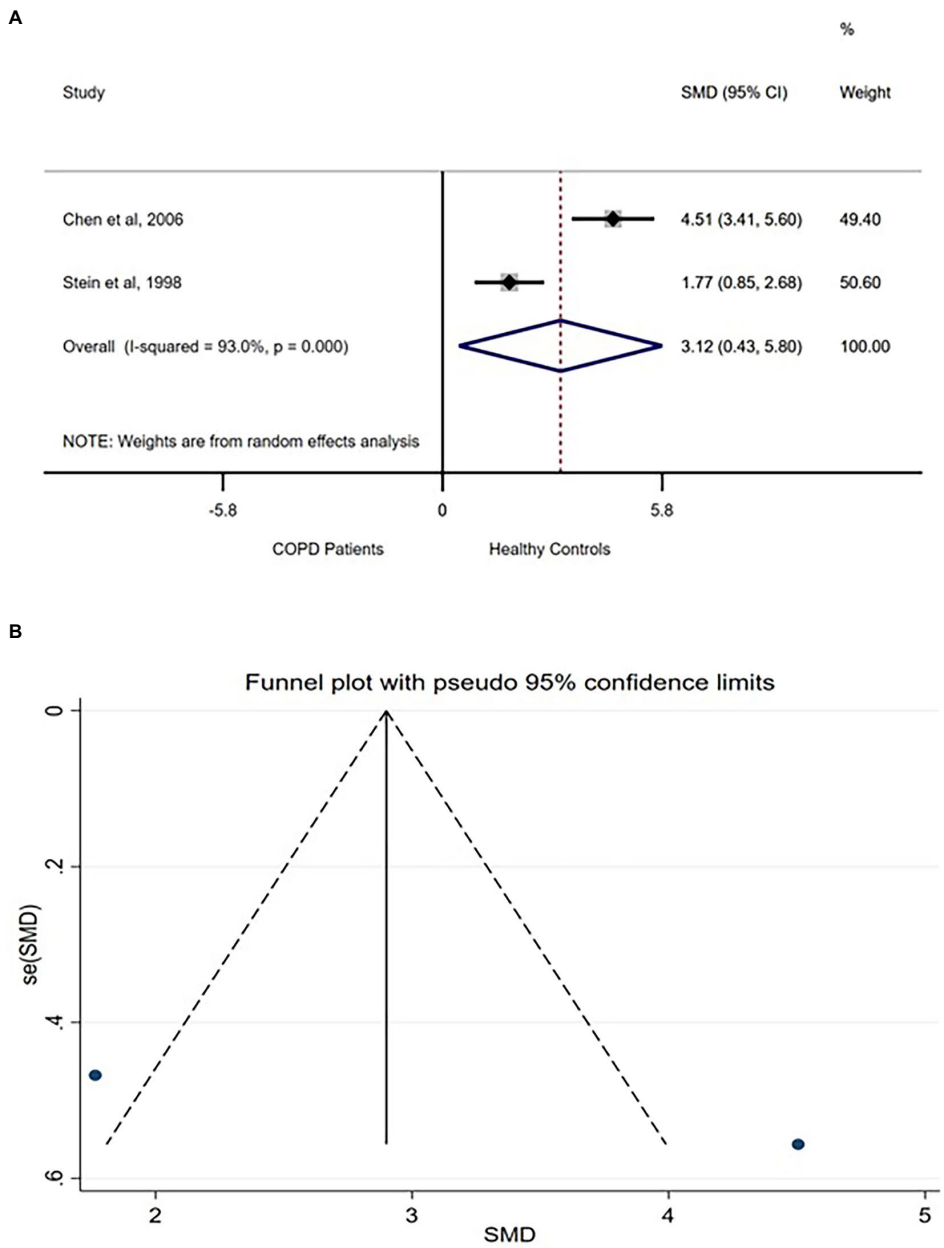
TP forest plot **(A)** showing the SMD and funnel plot **(B)** assessing the presence of publication bias.

Total power of the frequency bands physiologically indexes the combined influences of various controls including the sympathetic and parasympathetic autonomic nervous systems on the heart rhythm (6). Three studies compared TP between COPD patients and healthy controls (9, 10, 37). However, one (6) of the studies reported higher TP in acutely exacerbated COPD patients, while the other two studies reported lower TP in COPD patients. Because the analysis for SMD is not robust for difference in direction of difference, only the two studies (9, 10) were analyzed. A very large difference was found in TP between the groups [SMD (95% CI = 3.12) (0.43–5.80)] with a significantly high between-studies heterogeneity (Figure 4A). Funnel plot involving the two studies did not show any publication bias (Figure 4B).

#### Low frequency

Low frequency power is physiologically linked with baroreflex and parasympathetic modulation of heart rhythm under normal breathing condition (6). The low frequency power could be expressed in its natural form (LF, ms^2^) or in the normalized form (i.e., nuLF = LF/TP). Six studies compared LF between COPD patients and healthy controls (9, 19, 20, 31, 36, 37) with one study reporting higher LF in COPD patients (6). The 5 studies reporting lower LF in COPD patients were analyzed. Large difference was found in LF between the group [SMD (95% CI) = 1.82 (0.59–3.04)] with significantly high between-studies heterogeneity (Supplementary Figure S7A). Funnel plot showed not publication bias (Supplementary Figure S7B) and sensitivity test showed none of the analyzed studies exaggerated the effect size (Supplementary Figure S8).

#### High frequency (HF)

HF frequency power band is the respiratory band and reflects the effect of parasympathetic control on the heart rhythm corresponding to respiratory sinus arrythmia (6). HF is also expressed as absolute power (HF, ms^2^) or normalized power (i.e., nuHF = LF/TP). Six studies (9, 19, 20, 31, 36, 37) compared absolute HF between COPD patients and healthy controls of which 4 (9, 20, 31, 36) reported relatively higher HF in healthy controls while the 2 studies (19, 37) reported higher HF in COPD patients. Analysis of the 4 studies showed a large difference in HF between patients with COPD compared with healthy controls with significantly high between-studies heterogeneity (Supplementary Figure S9A). Funnel plot showed no publication bias (Supplementary Figure S9B). However, sensitivity test showed that the effect size is significantly exaggerated by the study by Carvalho et al. (20) (Supplementary Figure S10).

#### Low frequency–high frequency ratio

The ratio of LF/HF power has been classically translated as the measure of the balance between sympathetic and parasympathetic (vagal) controls on the heart rhythm. This was based on the assumption that the LF is solely contributed by the sympathetic cardiac rhythm control while the HF is mainly related to the parasympathetic, vagal control of the heart rhythm (6). However, studies have shown that the LF is also linked with parasympathetic control of the heart rhythm and the LF: HF, although important measure, does not translate purely to sympatho-vagal cardiac circle regulation (41, 42). Three studies compared LF:HF ratio between COPD patients and healthy controls of which 2 reported relatively higher (17, 25) LF:HF in COPD patients and the third study reported otherwise (19). A large difference in LF: HF (SMD (95%CI) = 1.46 (0.29–2.63)) was observed between the group with significantly high between-studies heterogeneity (Supplementary Figure S11A). Funnel plot showed no publication bias (Supplementary Figure S11B). Further, 2 studies compared normalized HF (nuHF) between COPD patients and healthy controls (11, 25). Large difference was found between the groups (SMD (95%CI) = 1.29 (0.11–2.47)) whereby COPD patients have higher nuHF compared with healthy controls. The between-studies heterogeneity was significantly high (Supplementary Figure S12) with no publication bias (Supplementary Figure S13).

### HRV non-linear indices in COPD

A single study reported compared HRV non-linear indices between patients with COPD and healthy controls (20). The authors compared the long-term (α2) and short-term (α1) fractal components of the detrended fluctuation analysis (DFA) which accesses the presence of fractal correlation in the NN intervals. The authors reported a significance difference in the short-term fractal components between the groups with lower α1 of the DFA found in COPD patients compared with healthy controls (0.9 ± 0.18 vs. 1.02 ± 0.18). This translates to impairment in fractal correlation in the dynamics of NN intervals in COPD patients compared with healthy controls.

## Discussion

We present here for the first time, a systematic review and meta-analysis of HRV in patients with COPD. Our study found supportive evidence that patients with COPD have a decrease in the HRV. COPD has a negative influence on almost all HRV parameters, reflecting the fact that COPD leads to a cardiac autonomic dysfunction, which may be explained by multiple underlying factors related to the disease. Although COPD severity likely plays a role in reduced HRV, this relationship still lacks further evidence.

### Sympathetic and parasympathetic modulation changes

Physiologically, the autonomic nervous system responds to a stressful situation by increasing sympathetic activity and decreasing parasympathetic activity, leading to a state of alertness (43). Interestingly, common chronic diseases such as cancer (44), obesity and metabolic syndrome (45), heart failure (46), cirrhosis (47), smoking (48), and hypertension (49), are associated with an activation of sympathetic and a decrease in parasympathetic nervous activity. In our study, we found that patients with COPD have a major reduction in all HRV parameters, including the LF band, which indexes the combined actions of the vagal and sympathetic controls of the heart, with a predominance of the sympathetic (50). At the same time, the LF:HF ratio was found to be increased, which indicates a sympathetic dominance (6). Thus, these findings reveal that COPD patients have decreased sympathetic and parasympathetic activity but remains with a predominance of sympathetic activity. In this context, sympathetic hyperactivity in these patients may be explained by several pathophysiological changes due to recurrent hypoxemia, hypercapnia, increased intrathoracic pressure swings due to airway obstruction, increased respiratory effort, systemic inflammation, or the use of betasympathomimetics (35).

However, under acute exacerbation the neural modulation of the cardiac rhythm may be altered, as evidenced in some studies, and interpretation of findings should be in the context of patients’ clinical condition. For instance, Kabbach et al. (51) found higher values of parasympathetic and global HRV, as well as lower values of complexity of the HR signal in AECOPD patients. Similarly, Zamarron et al. (37) revealed higher HF, LF:HF and total HRV power in exacerbated patients compared to stable ones and, Tseng et al. (33) in an emergency department have verified a greater increase in HF and decrease in LF:HF in those patients requiring admission compared to those discharged. Put together, because HRV indices are influenced by varied and diverse physiological pathways, interpretation of results may need to be clinically contextualized.

In terms of reduction in fractal correlates reported in COPD patients, this translates to reduced complexity and increased similarity in the N-N intervals. Physiologically, there is complex interplay between various organ systems in the body in response to an ever-changing environment. These interactions, aimed at maintaining homeostasis translates into higher complexity in physiological time series which may be reduced in patients with chronic disease which may have altered/reduced the ability to maintain a stable state. Indeed, previous studies have shown that reduced complexity in physiological time series may be associated with poorer prognosis in various settings (52–56).

### HRV and COPD severity

Some studies analyzed link between COPD severity and HRV changes. Incalzi et al. (16) studied patients with stable COPD and found a correlation between 24-h LF: HF and FEV_1_ (*r* = 0.321; *p* = 0.022). Consistent with these findings, Bedard et al. (18) also observed a correlation between the 24-h LF: HF ratio and FEV_1_ (*r* = 0.342; *p* = 0.028), which remained significant even after multivariate analysis. These results suggest that greater disease severity (lower FEV_1_ values) is accompanied by predominance of vagal modulation, given that a low LF:HF ratio indicates parasympathetic activity dominance (6). This may not be associated with the patients’ clinical condition however, it may be due to the vagal influence on airway narrowing (bronchoconstriction) (51).

Mazzuco et al. (28) showed a significant positive correlation between LF: HF and total gas volume (*r* = 0.53) and residual volume (*r* = 0.52), and negative correlation between the HF power and the total gas volume (*r* = −0.53). The latter association, suggests a relationship between decreased vagal modulation and increased total lung capacity, an index of static hyperinflation which is a marker of disease severity. Corroborating these findings, Corbo et al. (22) found positive associations between parasympathetic HRV parameters (rMSSD and HF) with FRC/TLC ratio. On the other hand, Camillo et al. (8) found that reduced HRV is not necessarily linked to COPD severity, estimated by FEV_1,_ and BODE index, but rather to the level of physical activity in daily life. Recently, Castello-Simões et al. (38) have demonstrated a dominance of the clinical status (acute or stable) instead of the severity of the disease on brain heart autonomic axis function assessed by HRV indices and heart rate recovery after exercise.

The relationship between COPD and HRV (and autonomic derangement) is further complicated by the increased risk of cardiovascular diseases that have been reported in patients with COPD. Specifically, patients with COPD have been shown to develop arrhythmia (e.g., atrial fibrillation), heart failure, pulmonary circulation, and arterial diseases, as well as ischemic heart disease, all of which may influence the autonomic regulation of the heart and conversely, HRV (57–59). Further, the increased risk of systemic inflammatory response in COPD patients has been linked with arteriosclerosis and myocarditis, which may also derange the autonomic nervous system’s physiological regulation of heart rhythm (60). Indeed, a recent study by Oyelade et al. showed that in patients with a high risk of arrhythmia, heart rate turbulence, which indexes the autonomic regulation of heart rhythm following premature ventricular complexes (PVCs), is more robust and may provide a more accurate alternative to HRV (61).

### Clinical implications and limitations

Reduced HRV has a powerful prognostic significance for cardiac events, including angina pectoris, myocardial infarction, coronary heart disease death, and congestive heart failure (62). In populations without known cardiovascular disease, low HRV is associated with a 32–45% increased risk of a first cardiovascular event (63). Similarly, a previous systematic review and meta-analysis showed reduced HRV indices in patients with cirrhosis reported overall HRV which may be linked with poorer prognosis (47). As shown here, COPD patients have an overall decrease in HRV parameters. This result may provide a physiological basis for the increased cardiovascular morbidity and mortality observed in COPD patients (64). In addition, COPD is also independently associated with cardiac arrhythmias, including atrial fibrillation/atrial flutter, non-sustained, and sustained ventricular tachycardia (65). This is another evidence suggesting that COPD patients are more susceptible to electrical disturbances and abnormalities of heart rhythm, which may predispose cardiac autonomic dysfunction. Additionally, HRV may have valuable prognostic value for other important COPD-related events, such as the risk of hospitalizations for exacerbation (33) and sudden death (66).

Considering the multifactorial basis of cardiac autonomic dysfunction, and the complex aetiology and clinical characteristics of COPD with multiple phenotypes and endotypes (e.g., frequent exacerbator, non-hyperinflator, emphysematous, overlap comorbidities, physical frailty) (67), the translation of the findings into real practice should be cautious. Traditionally, studies have established the cardiac autonomic profile at rest. However, a dynamic assessment and a reduced responsiveness to a stressor stimulus (physical exercise, postural change, and respiratory manoeuvres) may provide in-depth information and characterize numerous pathophysiological states.

This review has various limitations some of which are inherent to meta-analysis (68). Firstly, most of the included studies were cross-sectional, and the different measurement conditions of HRV parameters as well as heterogeneity of the populations studied possibly led to high inter- and intra-operator variability which is reflected in the high between-studies heterogeneity presented in the forest plots. However, the sensitivity test based on leave-one-out analysis showed not exaggerated effect. Further, while there are similarities between the study populations, they are not clinically identical. Also, although we found that HRV is reduced in COPD according to most studies, whether COPD severity and prognosis is linked with magnitude of HRV changes is still unclear. In addition, most of the studies determine severity criteria based on spirometry measurements, which can be a limitation for clinical applicability. An interesting perspective for future studies would be to associate HRV changes with the ABCD classification proposed by GOLD, which is based on information about the burden of symptoms and risk of exacerbation and has greater clinical applicability (69). In addition, the health status of controls was not sufficiently detailed in most of the studies, which may have influenced HRV. This may also have attenuated the magnitude of differences in HRV parameters when comparing COPD patients and controls. However, most of the included studies were homogeneous according to funnel plots and we computed the standardized mean difference (SMD) which is robust to some of these differences. Additionally, the final number of patients included in the meta-analysis is low, which may make it difficult to generalize the findings. Finally, the inclusion criteria allowed only non-classical HRV measurement algorithms, which may limit the scope of the results. Currently, wearable systems have been used to facilitate the capture of biosignals in clinical practice (60). However, the validity of these methods still needs to be extensively proven. In this review, included studies used mixture of analysis conditions, whereby some studies conducted HRV assessments in exercise conditions (17, 21) during non-invasive ventilation stimulus (19), and autonomic modulation (8), which deserves attention when generalizing these findings.

As a final point, it is important to highlight that in 2020 Catai et al. (70) have published a 30-item checklist to HRV procedures as crucial to dissemination of the HRV findings. The authors described the most common shortcomings and recommendations for solutions in HRV, which included the need for complete characterization of individuals and preparation for signal recordings, environmental condition, data collection method and standard devices, recording length and software, data processing and analysis details. Although it was not the focus of our study, it remains as an overall limitation in the interpretation of HRV studies and deserves to be considered in future studies for HRV to become useful in monitoring COPD patients.

## Conclusion

COPD patients have an overall decrease in HRV parameters which may translate as a significant breakdown in the coupling between the heart’s intrinsic pacemaker and extrinsic control mechanism such as the autonomic nervous (vagal) control system. Both sympathetic and parasympathetic cardiac modulation were decreased, but there is still a predominance of sympathetic activity. Available data does not allow the evaluation of the relationship between COPD severity and HRV reduction. Heterogeneity in methodology is a major limitation affecting clinical interpretation of HRV in COPD patients and should be prioritized by experts. The benefits of an HRV evaluation in assessing COPD should be studied further, given its potential as a simple, easily accessible technique that can be recorded and monitored remotely especially in this face of global pandemics.

## Author contributions

JA and TO: conception, design, data acquisition, analysis, interpretation, drafting for intellectually important content, and approval of final version. AAld, SAlg, IA, and AAls: data acquisition and approval of final version. AAlq, NA, LS, SAlr, EA, AH, RM, AAAlq, and AAla: analysis, interpretation, and approval of final version.

## Conflict of interest

The authors declare that the research was conducted in the absence of any commercial or financial relationships that could be construed as a potential conflict of interest.

## Publisher’s note

All claims expressed in this article are solely those of the authors and do not necessarily represent those of their affiliated organizations, or those of the publisher, the editors and the reviewers. Any product that may be evaluated in this article, or claim that may be made by its manufacturer, is not guaranteed or endorsed by the publisher.
