# Supplementary material for: A systematic review and meta-analysis of heart rate variability in COPD
Source: Front Cardiovasc Med. 2023 Feb 17;10:1070327. doi: 10.3389/fcvm.2023.1070327 (PMC9981678; doi:10.3389/fcvm.2023.1070327)
Supplement: Supplementary file 1 [file Data_Sheet_1.PDF]

Supplemental materials

A systematic review and meta-analysis of heart rate variability in COPD

Jaber S Alqahtani[1], Abdulelah M Aldhahir[2], Saeed M Alghamdi[3], Shouq S Al Ghamdi[4], Ibrahim A AlDraiwiesh[1], Abdullah S Alsulayyim[2,3], Abdullah S Alqahtani[1], Nowaf Y Alobaidi[5,6], Lamia Al Saikhan[7], Saad M AlRabeeah[1], Eidan M. Alzahrani[8], Alessandro D Heubel[9], Renata G Mendes[9], Abdullah A Alqarni[10], Abdullah M. Alanazi[11,12], Tope Oyelade[13]

1 Department of Respiratory Care, Prince Sultan Military College of Health Sciences, Dammam 34313, Saudi Arabia;

2 Respiratory Therapy Department, Faculty of Applied Medical Sciences, Jazan University, Jazan 45142, Saudi Arabia

3 National Heart and Lung Institute, Imperial College London, London SW7 2BX, UK; Respiratory Care Program, Clinical Technology Department, College of Applied Health Science, Umm Al Qura University, Makkah 21955, Saudi Arabia.

4 Anesthesia Technology Department, Prince Sultan Military College of Health Sciences, Dammam 34313, Saudi Arabia.

5 Institute of Inflammation and Ageing, University of Birmingham, Birmingham, UK

6 Respiratory Therapy Department, King Saud bin Abdulaziz University for Health Sciences, Alahsa, Saudi Arabia

7 Department of Cardiac Technology, College of Applied Medial Sciences, Imam Abdulrahman Bin Faisal University, 2835 King Faisal Street, Dammam 34212, Saudi Arabia.

8 Physical Therapy Department, Prince Sultan Military College of Health Sciences, Dammam 34313, Saudi Arabia.

9 Cardiopulmonary Physiotherapy Laboratory, Department of Physical Therapy, Federal University of São Carlos, SP, Brazil.

10 Department of Respiratory Therapy, Faculty of Medical Rehabilitation Sciences, King Abdulaziz University, Jeddah 21589, Saudi Arabia.

11 Department of Respiratory Therapy, College of Applied Medical Sciences, King Saud bin Abdulaziz University for Health Sciences, Riyadh, Saudi Arabia, 14611.

12 King Abdullah International Medical Research Center, Riyadh, Saudi Arabia, 14611.

13 UCL Institute for Liver and Digestive Health, London, United Kingdom

*Corresponding author

Dr Jaber S. Alqahtani, Department of Respiratory Care, Prince Sultan Military College of Health Sciences,

Dammam 34313, Saudi Arabia, alqahtani-jaber@hotmail.com

Supplemental materials

Table S1. ECG recording and HRV analysis techniques of included studies.

Study	Aim	Country	Sample size (Male) (COPD/Control)	Age Mean ±SD or Median (IQR) (COPD/Control)	COPD GOLD severity (based on FEV1%)	Equipment used	ECG Duration	ECG Sampling Frequency	Software	Indices measured	Significant indices	Conclusion/Findings
Incalzi et al, 2009 (1)	To assess the relationship between autonomic dysfunction and COPD severity	Italy	55 (46)	69.1 ± 7.7	NR	Portable three-channel tape recorder	24 hours	128 Hz.	Marquette 8000 T system	TP, VLF, LF, HF, LF/HF, nuLF, nuHF, nuLF/nuHF	VLF Power, LF n.u, HF n.u., and LF n.u./HF n.u. ratio	Autonomic control measured by HRV indices deteriorates as COPD worsens. Also, HRV reduced significantly with neuropsychological tests (CDL) of patients with COPD.
Bartels et al. 2003 (2)	To evaluate cardiac autonomic modulation in patients with COPD during peak exercise	USA	53 (27)/14(7)	63 ± 10/60 ± 8	NR	Marquette Max-1; Marquette Medical Systems; Milwaukee, WI	NR	200 Hz.	NR	lnHF, lnLF, nuHF, nuLF, LF/HF	nuLF, LF/HF	The sympathetic to parasympathetic cardiac modulation decreases in patients with COPD at rest and during maximal volitional exercise compared with healthy controls
Bedard et al, 2010 (3)	To compare in COPD patients and healthy controls during normal daily life and to evaluate the influence of anticholinergic andβ-adrenergic medications on HRV in COPD patients	Canada	41 (28)/19(14)	67 ± 7	NR	NR	24 hours	NR	NR	SDNN, SDANN, rMSSD, pNN50, LF, HF, LF:HF	LF:HF	COPD patients have a reduced global HRV compared with healthy subjects during normal daily life. This decreased HRV correlates with disease severity and does not seem to be influenced by anticholinergic or adrenergic medications.
Borghi-Silva et al, 2008 (4)	To evaluate the acute effects of bi-level positive airway pressure (BiPAP) on heart rate variability (HRV) of stable chronic obstructive pulmonary disease patients (COPD).	Brazil	19 (19)/8(8)	69 ± 8	GOLD-3 (FEV1 = 35 ± 9)	Polar T31 transmitter, Polar Electro, Kempele, Finland + cardiac monitor Ecafix TC500, Sã˜o Paulo,SP, Brazi	10 minutes		Developed in-house based on MatLab	SDNN, rMSSD, LF, HF, nuLF, nuHF, LF:HF	LF, nuLF, nuHF, LF:HF	Sympathetic and parasympathetic neural control of heart rate is altered in COPD patients. The improvement of ventilation caused by i-level positive airway pressure (BiPAP) is associated with the reduction of cardiac vagal activity in stable moderate-to-severe COPD patients.

Supplemental materials

Camillo et al, 2008 (5)	To study the relationship between HRV and different disease characteristics which indicate disease severity and the degree of pulmonary, muscular, and functional impairment in patients with COPD, such as the BODE index and its variables, exercise capacity, respiratory and peripheral muscle force, body composition, and level of physical activity in daily life	Brazil	31 (16)	66 ± 8	FEV1% (46 ± 15)	Polar S810i monitor, Polar Electro OY, Kempele, Finland	20 minutes (supine, 10 min/tilt, 10 min)	1000 Hz.	NR	SDNN, VLF, LF, HF, TP, LF:HF	SDNN, nuLF, nuHF	Cardiac autonomic function measured by HRV of patients with COPD is not related to disease severity but mainly to the level of physical activity in daily life.
Carvalho et al, 2011 (6)	To evaluate short- and long-term fractal exponents of HRV in COPD subjects	Brazil	15 (10)/15(8)	73.93 ± 6.61/ 68.73 ± 7.27	FEV1% (51.69 ± 16.32)	Polar S810i monitor, Polar Electro OY, Kempele, Finland	5 minutes	1000 Hz.	HRV analysis	SDNN, rMSSD, LF, HF, LF:HF, DFA (α1, α2, α1:α2)	SDNN, LF, HF, rMSSD, α1	COPD subjects present reduced short-term fractal correlation of HRV, indicating sympathetic and parasympathetic activity reduction.
Chang et al, 2011 (7)	To noninvasively investigate cardiac autonomic modulation in patients with severe COPD during heavy and very heavy exercise at and above the CP.	USA	9 (6)	60.2 ± 6.9	NR	Polar RS800CX, Polar Electro Oy, Kempele, Finland	512 Beats (pre- and post-exercise each)	NR	NR	SDNN, rMSSD, NN50, TINN, VLF, LF, HF, LF:HF	rMSSD, TINN, LF:HF, nuLF, nuHF	Autonomic balance is shifted toward parasympathetic tone dominance in patients with severe COPD.
Chen et al, 2006 (8)	To examine the relationship between the derangements in the cardiac autonomic nervous function and the oxygenation status or degree of airflow obstruction in COPD patients by using HRV analysis	Taiw an	30(25)/1 8(15)	69.6±6.5/ 64.8±9.0	FEV1% (50.5±1 9.9)	CG monitor (SpaceLab 90621A Monitor,Spacel abs Inc., Redmond, WA)	15 minutes	NR	Mathcad11, Mathsoft Inc., Cambridge, MA	SDNN, CVNN, HF, LF, nuHF, nuF, TP, LF:HF	SDNN, CVNN, HF, LF, TP	The worse the oxygenation status of the patients is, the more cardiac vagal and lesser cardiac sympathetic activities the patients have
Corbo et al, 2013 (9)	To assess whether HRV at rest and during physical activity, is influenced by the severity of the COPD and whether the influence is related to systemic inflammation.	Italy	30(25)	65.92 ± 9.73	FEV1% (47.72± 18.32)	NR	NR	NR	NR	SDNN, rMSSD, TP, VLF, LF, HF, LF:HF	SDNN, TP, VLF	COPD patients with moderate and severe disease had an abnormal cardiac autonomic modulation which was related to both systemic inflammation and lung function impairment
Goulart et al, 2016 (10)	To assess if alterations in respiratory muscle strength may affect cardiac autonomic modulation in COPD patients	Brazil	10(8)	61.2 ± 6.7	FEV1% (31.9±1 3.6)	Polar S810i, Kempele, Finland	14 minutes		Kubios HRV analysis software (version 2.2, Matlab Kuopio, Finland)	SDNN, rMSSD, RRTri, nuLF, nuHF, LF:HF, ApEn, SampEn	nuLF, nuHF, LF:HF, ApEn, SampEn	COPD patients with impaired respiratory muscle strength showed marked sympathetic modulation and a reduced parasympathetic response and reduced HRV complexity.

Supplemental materials

Gunduz et al, 2009 (11)	To evaluate the HRV and HRT variables in COPD patients	Turkey	25(22)/25(19)	63 ± 7/60 ± 8	FEV1% (44 ± 15)	Del Mar Reynolds Pathfinder Holter system.	24 hours	NR	Del Mar Reynolds Pathfinder Holter system (software).	SDNN, SDNNI, SDANN, rMSSD, NN50 count, pNN50	SDNN, SDNNI, SDANN, rMSSD, NN50 count, pNN50	HRV is significantly different in COPD patients compared with healthy controls.
Surulichamy et al 2017 (12)	To access the HRV in patients with COPD and to compare with normal individuals.	India	30(23)/30(23)	43.72±4.42/43.72 ±4.34	NR	Computerized 8 channel polygraph (Model: Physiopac, Medicaid Systems, Chandigarh, India)	NR	NR	Computerized 8 channel polygraph (Model: Physiopac, Medicaid Systems, Chandigarh, India)	NN50 count, pNN50, rMSSD, nuHF, nuLF, LF:HF	rMSSD, NN50 count, pNN50, nuHF, nuLF, LF:HF	COPD patients have an autonomic imbalance suggestive of an increased sympathetic tone or decreased parasympathetic tone. Sympathetic overactivity may lead to cardiovascular disease development in patients with COPD
Leite et al, 2015 (13)	To assess the link between resting heartrate variability (HRV) indexes with aerobic physiological variables obtained at a maximal exercise test in patients with COPD	Brazil	36(22)	63 (59–70)	FEV1%(46 (35.4–63.7))	Polar S810i (PolarElectro, Kempele, Finland)	256 RR intervals (of 20 minutes)	NR	Kubios HRV software (version 2.0)	SDNN, rMSSD, LF, HF, nuLF, nuHF, LF:HF, SD1, SD2	SDNN, rMSSD, LF, HF, nuLF, nuHF, LF:HF, SD1, SD2	HRV indexes at rest correlate with aerobic capacity in COPD patients.
Lu et al, 2016 (14)	To access the clinical significance of the cross-spectral measures of ECG and nostril airflow signals in COPD patients	Taiwan	23(19)/23(20)	81 (76–83)/ 77 (76–78)	FEV1%(66.9 (55.6–90.6))	Multichannel Recorder (Biopac MP35, Biopac Systems, Inc., Goleta, CA)	512 RR Intervals (of 15 minutes)	500 Hz.	Mathcad 13 software (Mathsoft Inc., Cambridge, MA).	SDNN, CVNN, rMSSD, TP, VLF, LF, HF, LF:HF, nuVLF, nuLF, nuHF	SDNN, CVNN, rMSSD, TP, HF, LF, HF, LF:HF	Cross-spectral analysis of ECG and nostril airflow provides information about the cardiovascular-related functions of COPD patients.
Mazzuco et al, 2015 (15)	Ta access whether impairment of static lung volumes and lung diffusion capacity could be related to HRV indices in patients with moderate to severe COPD	Brazil	16 (16)	66.3±8.4	FEV1%(53.9±19.7)	Polar T31 transmitter (Polar Electro, Kempele, Finland)	≥256 RR Intervals	NR	Kubios HRV® version 2.1 software (Matlab, Kuopio, Finland).	Mean RR, SDNN, rMSSD, nuLF, nuHF, LF:HF, SD1, SD2, DFA (α1, α2), ApEn	rMSSD, LF, nuLF, nuHF, LF:HF, SD1, SD2, DFA (α1)	Greater lung function impairment was related to poorer heart rate variability in patients with COPD
Mendes et al, 2011 (16)	To analyse heart rate (HR), blood pressure and heart rate variability in COPD patients undergoing FVC testing	Brazil	29 (29)	72±8	53.1±29.2	Polar S810i frequency meter (Polar Electro, Kempele, Finland)	≥256 RR Intervals	1000 Hz.	HRV Analysis Software (Biosignal Analysis and Medical Imaging Group, University of Kuopio, Finland)	rMSSD, VLF, LF, HF, nuLF, nuHF, LF:HF	NS	The FVC test influences the behaviour of COPD patient HR without changing autonomic control or BP.

Supplemental materials

Pan et al, 2018 (17)	To access whether indoor particulate matter and black carbon may affect the HRV/HR in patients with COPD.	China	43(40)	71.49 ± 6.40	FEV1% (< 80%)	12-channel Holterrecorder (model MGY-H12; DM Software Inc., USA)	24 hours	NR	Holter System, version12.net; DM Software Inc., USA	SDNN, SDANN, rMSSD, pNN50, LF, HF, LF:HF	SDNN, rMSSD, pNN50, LF, HF, LF:HF	Exposures to size-fractioned indoor particulate matter and black carbon were associated with significant reductions in HRV indices and increases in HR in COPD patients
Reis et al, 2010 (18)	To evaluate the influence of respiratory muscle strength on the magnitude of respiratory sinus arrhythmia.	Brazil	10(10)/9(9)	69 ± 9	FEV1% (41 ± 11)	TC 500 (Ecafix, São Paulo, SP, Brazil)	Various*	NR	Developed in-house based on MatLab	SDNN, rMSSD, VLF, LF, HF, LF:HF, nuLF, nuHF	SDNN, rMSSD, LF, HF	Patients with COPD show impaired sympathetic-vagal balance at rest and show reduced cardiac autonomic control of heart rate linked with inspiratory muscle weakness.
Sima et al 2017 (19)	To assess the test-retest reliability of HRV measurement from short-term ECG recording performed during spontaneous breathing in individuals with moderate-to-severe COPD.	Canada	13 (8)	63 ± 6	FEV1% (46 ± 16)	SphygmoCor® CPV device (AtCor Medical, Inc., USA).	5 minutes	NR	NR	SDNN, rMSSD, LF, HF, TP, LF:HF	SDNN, rMSSD, LF, HF, TP, LF:HF	HRV measurements showed overall moderate-to-substantial reliability during spontaneous breathing in COPD population.
Stein et al, 1998 (20)	To determine if HRV is decreased or reflects severity in COPD and whether HRV is affected by PiZ α1-antitrypsin deficiency.	USA	13(NR)/13(NR)	42 ± 5	NR	Holter recorders (Marquette Series 8500; Milwaukee)	24 hours	NR	Marquette SXP Laser Holter Scanner (Software version 5.8)	Mean NN, SDNN, SDANN, SDNNI, rMSSD, pNN50, TP, LF, HF, VLF, ULF, LF:HF	SDNN, SDANN, TP, VLF, ULF	PiZ α1-antitrypsin deficiency COPD is associated with abnormal cardiac autonomic modulation
Tseng et al, 2018 (21)	To noninvasively evaluate cardiac autonomic modulation in patients with COPD, during acute exacerbation	Taiwan	33(32)	77.1 ± 1.6	GOLD stage 2 (16) GOLD stage 3 (17)	Del Mar Avionics ECG scanner (model 500)	5 minutes	245 Hz.	Developed in-house based on MatLab	TP, lnHF, nuHF, lnLF, nuLF, lnVLF, LF:HF	TP, nuHF, nuLF, LF:HF	Patients with AECOPD requiring admission after ED treatment had a greater increase in HF% and greater decrease in LF/HF ratio compared to those discharged
Tukek et al, 2003 (22)	To assess the possible effect of diurnal variability of heart rate on the development of arrhythmias in patients with COPD	Turkey	41(39)/32(27)	59±8.5/57±11	FEV1% (40±16)	Marquette SEER solid-state recorder (Marquette Electronics, Milwaukee, WI, USA)	24 hours	NR	Marquette Electronics Series 8000 Holter Analysis System Version 5.8, 1-Sept-92	SDNN, SDANN, SDNNI, rMSSD, pNN50, lnTP, lnLF, lnHF, LF:HF	NR (due to stratification by presence or absence of arrhythmia)	COPD patients with arrhythmia have circadian HRV disturbances such as unchanged night-time parasympathetic tone and disturbed sympatho-vagal balance in favor of the sympathetic system all day long, which may explain the increased frequency of arrhythmia
Gestel et al, 2011 (23)	To investigate if cardiac autonomic dysfunction plays a role in HRQL in patients with COPD	Germany	60(23)	65.2±7.7	FEV1% (46.58± 18.53)	3-channel ECG recording (Nexus-10, TMS International BV, The Netherlands)	5 minutes	NR	NR	Mean RR, SDNN, rMSSD, LF, nuLF, HF, nuHF, LF:HF	rMSSD, nuHF, LF:HF	Resting parasympathetic tone is independently associated with HRQL and emphasizes the role of cardiac autonomic dysfunction on HRQL in patients with COPD

Supplemental materials

Vanzella et al, 2018 (24)	To evaluate autonomic modulation in individuals with and without COPD	Brazil	43(NR)/31(NR)	66.37 ± 8.27/63.25 ± 7.13	FEV1% (54.79 ± 21.04)	Polar S810i (PolarElectro, Kempele, Finland)	1000 RR intervals (of 30 minutes)		Kubios HRV version 2.0 software (Kubios Oy; Kuopio, Finland)	SDNN, rMSSD, LF, nuLF, HF, nuHF, LF:HF	SDNN, rMSSD, LF, HF	COPD patients show a reduction in both sympathetic and parasympathetic activity, associated with decreased complexity of autonomic nervous system function.
Volterrani et al, 1994 (25)	To evaluate the presence of autonomic dysfunction in patients with COPD compared with normal population using HRV	Italy	31(31)/32(32)	55 ± 10/NR(age-matched)	FEV1% (52 ± 8.3)	ECG respiratory monitor (Kontron)	NR	1000 Hz.	NR	Mean RR, SDRR, CVNN, nuLF, nuHF, LF:HF	SDNN, nuHF	Patients with COPD have abnormalities of ANS function, specifically a depressed HRV response to sympathetic and vagal stimuli
Zamarron et al, 2014 (26)	To analyse heart rate variability in COPD patients under stable condition and during acute exacerbation episodes (AECOPD)	Spain	23(23)/8(8)	69.6 ± 7.3/68.6 ± 4.9	NR	NR	2 minutes	4 Hz	RHRV software	TP, LF, HF, LF:HF	TP, LF, HF, LF:HF	AECOPD patients exhibited signs of increased autonomic activity compared with stable COPD patients and control
Castello-Simões wt al. 2021 (27)	To investigate the brain-heart autonomic axis function across different clinical status and severity of COPD	Brazil	77 (50)	65.5± 8	Gold 1-4	Polar S810i system telemetry	10 minutes	NR	Kubios® HRV analysis software (MATLAB)	Mean RR, STDRR, RMSSD, RRtri, HF, LF, SD1, SD2, ApEn, SampEn	RMSSD, SD1, HF	Clinical status (AECOPD or stable) was more dominant than the severity on the brain-heart autonomic axis function

Supplemental materials

Table S2. Search strategy for Medline and Embase databases.

Ovid MEDLINE(R) ALL <1946 to Aug 10, 2022>	
1	exp Autonomic Nervous System/ or heart rate variability.mp. 163292
2	HRV.mp. 13604
3	Chronic Obstructive Lung Disease.mp. or exp Pulmonary Disease, Chronic Obstructive/ 65811
4	Chronic Obstructive Pulmonary Diseases.mp. or exp Pulmonary Disease, Chronic Obstructive/ 64008
5	COPD.mp. 54283
6	Chronic Obstructive Pulmonary Disease*.mp. [mp=title, abstract, original title, name of substance word, subject heading word, floating sub-heading word, keyword heading word, organism supplementary concept word, protocol supplementary concept word, rare disease supplementary concept word, unique identifier, synonyms] 57393
7	HRV*.mp. [mp=title, abstract, original title, name of substance word, subject heading word, floating sub-heading word, keyword heading word, organism supplementary concept word, protocol supplementary concept word, rare disease supplementary concept word, unique identifier, synonyms] 14859
8	1 or 2 or 7 166505
9	3 or 4 or 5 or 6 97835
10	8 and 9 376
11	limit 10 to english language 351
12	limit 11 to "reviews (best balance of sensitivity and specificity)" 61
13	11 not 12 290
14	limit 13 to case reports 5
15	13 not 14 285
16	limit 15 to editorial 2
17	15 not 16 283
18	limit 17 to letter 5
19	17 not 18 278
Embase Classic+Embase <1947 to 2022 July 10>	
1	heart rate variability.mp. or exp heart rate variability/ 39925
2	HRV.mp. 20359
3	HRV*.mp. [mp=title, abstract, heading word, drug trade name, original title, device manufacturer, drug manufacturer, device trade name, keyword heading word, floating subheading word, candidate term word] 22384
4	1 or 2 or 3 44764
5	Chronic Obstructive Lung Disease.mp. or exp chronic obstructive lung disease/ 158473
6	Chronic Obstructive Pulmonary Disease.mp. or exp chronic obstructive lung disease/ 167238
7	COPD.mp. 102049

Supplemental materials

8 COPD*.mp. [mp=title, abstract, heading word, drug trade name, original title, device manufacturer, drug manufacturer, device trade name, keyword heading word, floating subheading word, candidate term word] 102419

9 Chronic Obstructive Pulmonary Disease*.mp. [mp=title, abstract, heading word, drug trade name, original title, device manufacturer, drug manufacturer, device trade name, keyword heading word, floating subheading word, candidate term word] 84224

10 5 or 6 or 7 or 8 or 9 185446

11 4 and 10 526

12 limit 11 to english language 502

13 limit 12 to human 474

14 limit 13 to conference abstracts 159

15 13 not 14 315

16 limit 15 to "reviews (best balance of sensitivity and specificity)" 56

17 15 not 16 259

18 limit 17 to (chapter or conference paper or "conference review" or editorial or letter or note or "review") 24

19 17 not 18 235

20 limit 19 to (meta analysis or "systematic review") 1

21 19 not 20 234

Supplemental materials

Table S3. Details of the quality assessment of the included studies.

First author	Population representative	Sample size adequate	Confounders	Statistical analysis	Missing data	Methodology of the outcome	Objective assessment	OVERALL (0-3, higher score = lower risk of bias)
Camillo et al, 2008	2	2	3	2	3	2	3	2.4
Incalzi et al. 2009	3	2	3	3	2	2	3	2.5
Gunduz et al. 2009	3	2	0	3	3	2	3	2.2
Chang et al, 2011	3	1	0	2	3	3	3	2.1
Zamarron et al. 2014	2	2	0	2	2	3	3	2
Stein et al. 1998	3	1	3	2	2	3	3	2.4
Bartels et al. 2003	3	3	3	3	2	3	3	2.8
Chen et al. 2006	2	1	3	3	3	2	3	2.4
Borghi-Silva et al 2008	2	1	3	3	3	3	3	2.5
Lu et al. 2016	2	2	2	3	3	3	3	2.5
Tukek et al ,2002	0	0	0	0	2	1	1	0.57
Carvalho et al, 2011	0	0	1	2	0	1	2	0.85
Bedard et al , 2010	1	0	1	1	1	1	1	0.85
Mazzuco et al, 2015	1	0	0	1	3	2	1	1.14
Goulart et al 2016	0	0	1	1	2	1	2	1
Sima et al, 2017	1	0	2	1	2	1	2	1.29
Pan et al, 2018	1	1	2	1	0	1	2	1.14
Mendes et al. 2011	1	1	1	2	2	2	3	1.7
Surulichamy et al. 2016	1	1	1	2	3	2	3	1.86
Corbo et al. 2013	2	1	2	3	3	3	3	2.6
Gestel et al. 2011	2	1	1	3	3	3	3	2.3
Tseng et al. 2018	1	1	2	2	3	2	3	2.14
Volterrani et al. 1994	2	1	1	2	3	2	3	2.14
Vanzella et al.2018	1	1	2	2	3	2	3	2.14
Leite et al. 2015	1	1	1	2	3	2	3	1.85
Castello-Simões wt al.	2	2	3	2	3	2	3	2.4
Reis et al. 2010	1	1	1	2	3	2	3	1.85

Supplemental materials

Table S4. Definitions and units of included HRV indices

HRV Indices	Units	Descriptions
NN intervals.	ms	Time difference between consecutive normal QRS complexes of an ECG recording.
SDNN	ms	Standard deviation of NN intervals.
cSDNN	ms	Corrected SDNN is the standard deviation of NN interval that have been corrected for heart rate. It is calculated as SDNN/$e^{-(HR/58.8)}$
SDANN	ms	Standard deviation of the average NN intervals for each 5-minute segments deduced from a 24-hour ECG recording.
SDNN Index	ms	Mean of standard deviations of all NN intervals for each 5-minute segments deduced from a 24-hour ECG recording.
pNN50	%	Percentage of successive NN intervals that vary by more than 50ms.
RMSSD	ms	Root mean square of differences in successive NN interval.
TINN	ms	Width of the base of a computed RR interval histogram.
TP		Variance of entire NN interval of either 5 minutes (short) or 24-hour (long) ECG recording.
VLF	ms^2	Power of the frequency band between 0.0033-0.04 Hz.
LF	ms^2	Power of the frequency band between 0.04-0.15 Hz.
HF	ms^2	Power of the frequency band between 0.15-0.4 Hz.
LF/HF		Ratio of LF power to HF power.
SD1	ms	The length of the line or standard deviation perpendicular to the line of identity of a Poincare plot
SD2	ms	The length of the line or standard deviation parallel to the line of identity of a Poincare plot
ApEn (Approximate Entropy)		Measure of irregularity and complexity of a series of NN intervals.
SampEn (Sample Entropy)		Measure of irregularity and complexity of a series of NN intervals.
DFA α1 (Short-term scaling exponent)		Measure of short-term fractal-like fluctuations of inter-beat intervals.
DFA α2 (Long-term scaling exponent)		Measure of long-term fractal-like fluctuations of inter-beat intervals.

ECG, Electrocardiograph; NN Interval, time lapse between consecutive QRS complexes of ECG recording; SDNN, Standard deviation of NN intervals; ms, millisecond; SDANN, Standard deviation of the average NN intervals for each 5-minute segments deduced from a 24-hour ECG recoding; pNN50, Percentage of successive RR intervals that vary by more than 50ms; RMSSD, Root mean square of differences in successive NN interval; TINN, Triangular Interpolation of the NN intervals' histogram; TP, Total Power; VLF Very Low Frequency; LF, Low Frequency; HF, High Frequency; LF/HF, Ratio of LF to HF; SD1, Poincare plot Standard Deviation perpendicular to the line of identity; SD2, Poincare plot Standard Deviation along the line of identity; ApEn, Approximate Entropy; SampEn, Sample Entropy; DFA α1, Short-term fluctuation of Detrended Fluctuation Analysis; DFA α2, Long-term fluctuation of Detrended Fluctuation Analysis; ms, millisecond.

Supplemental materials

Supplemental materials

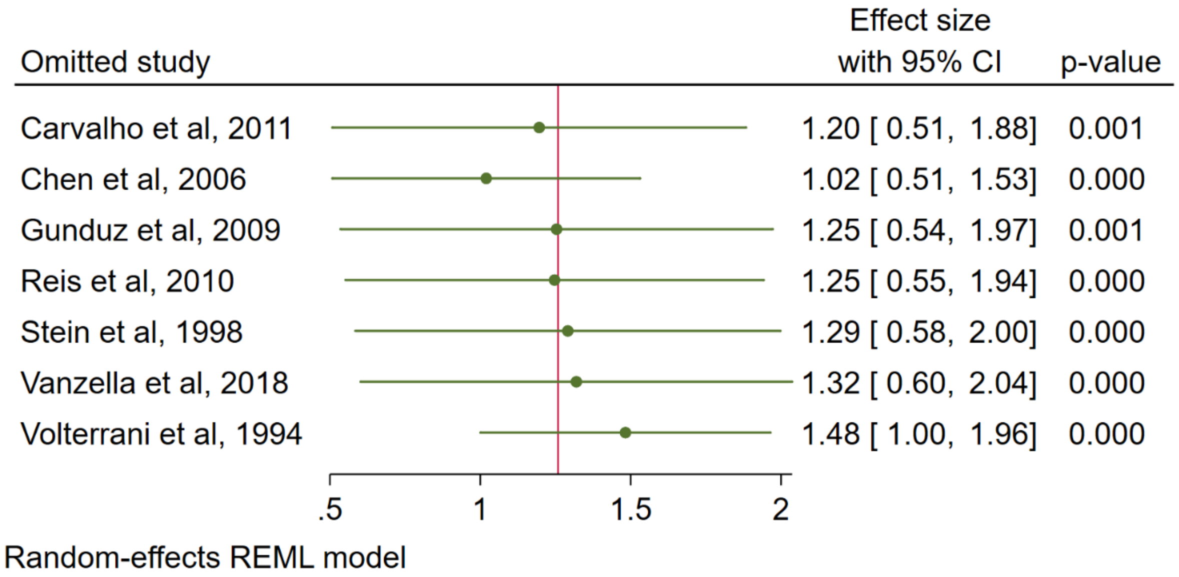

Figure S1. Leave-one-out sensitivity analysis to test whether any of the included studies introduced an exaggerated effect size to the computed SDNN.

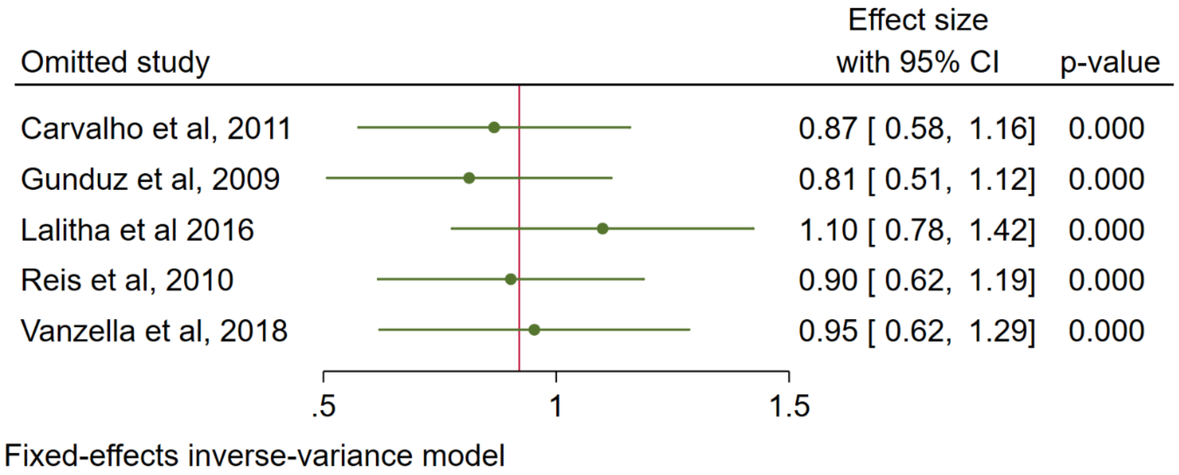

Figure S2. Leave-one-out sensitivity analysis to test whether any of the included studies introduced an exaggerated effect size to the computed rMSSD.

Supplemental materials

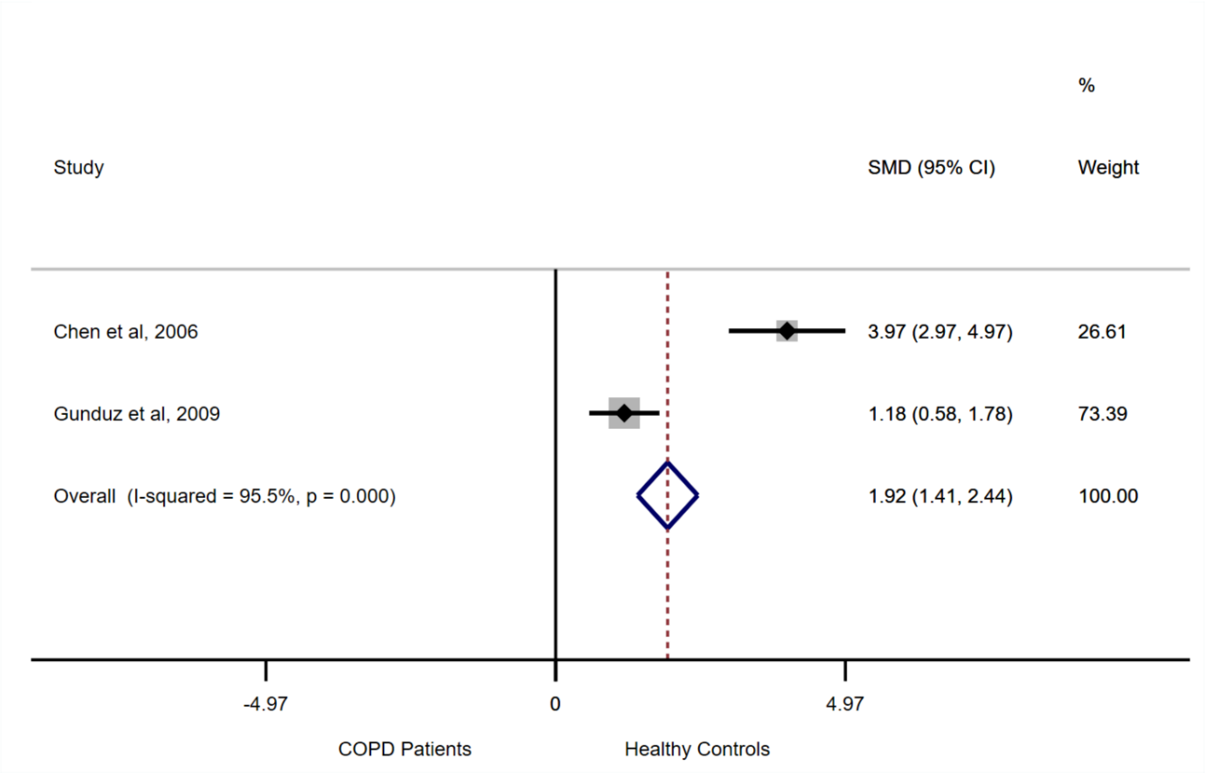

Figure S3. SDNNI

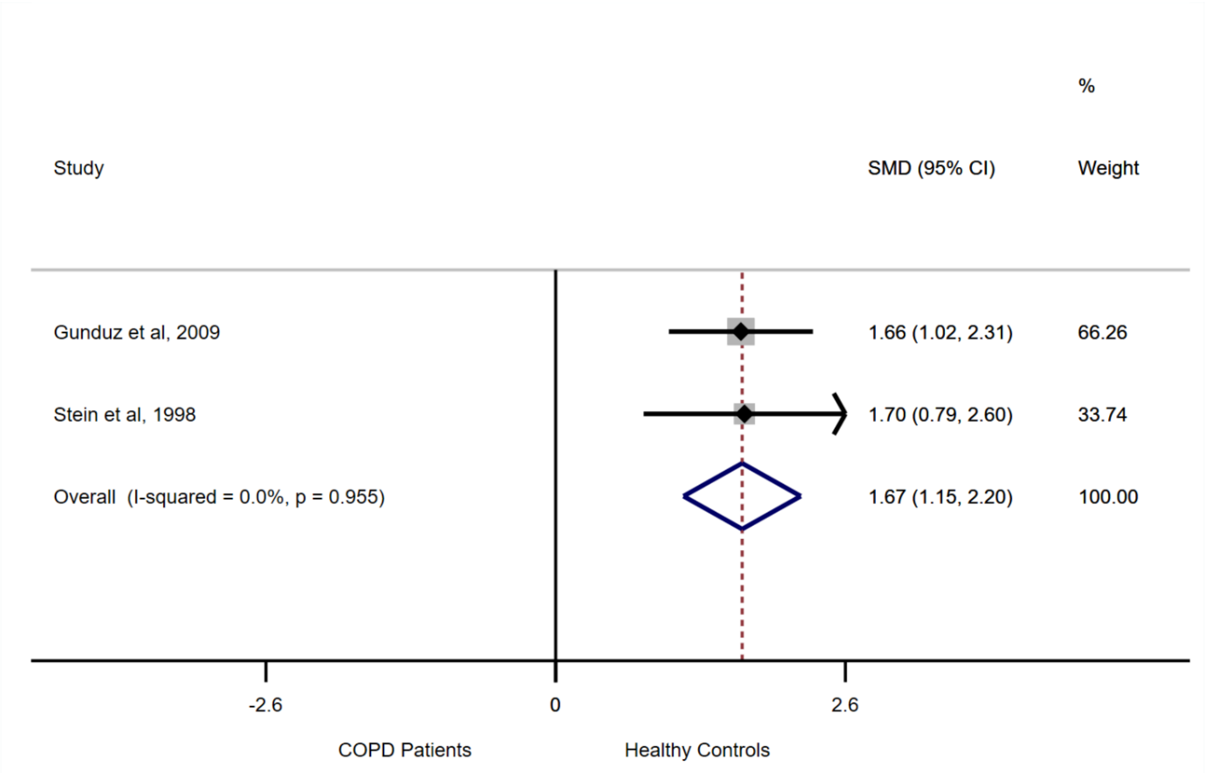

Figure S4. SDANN

Supplemental materials

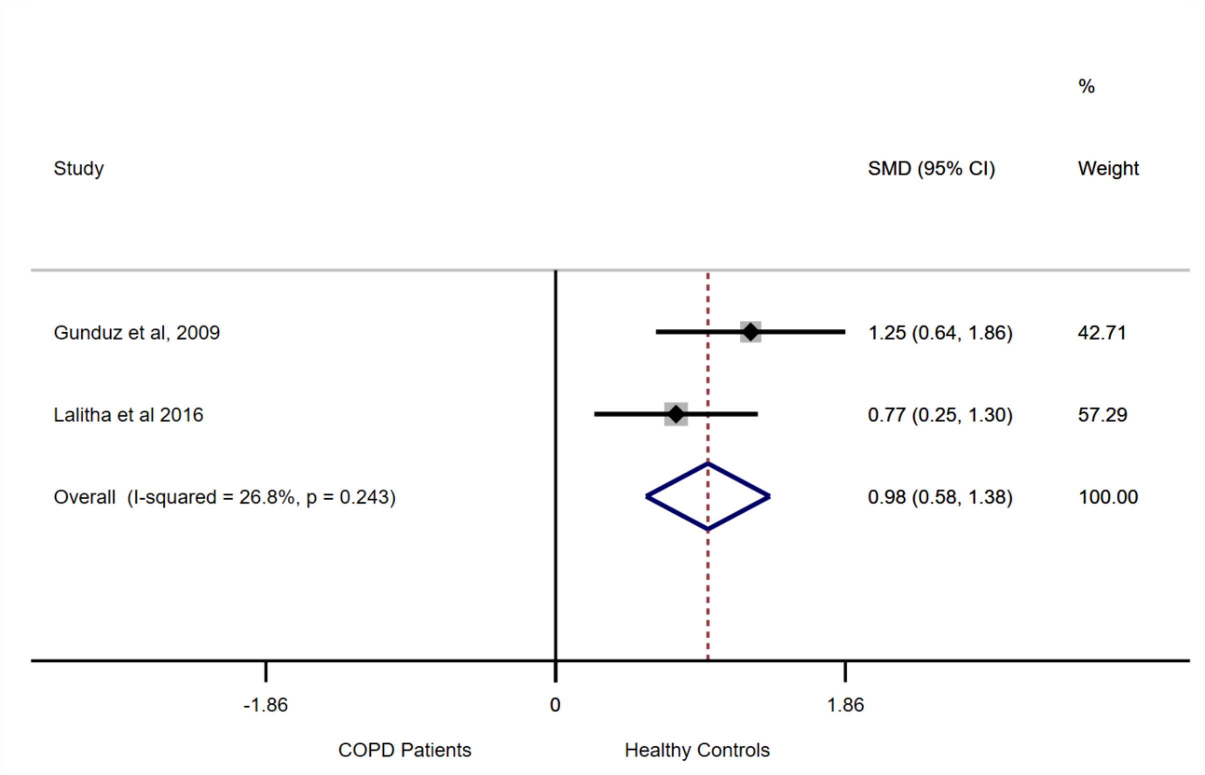

Figure S5. NN50

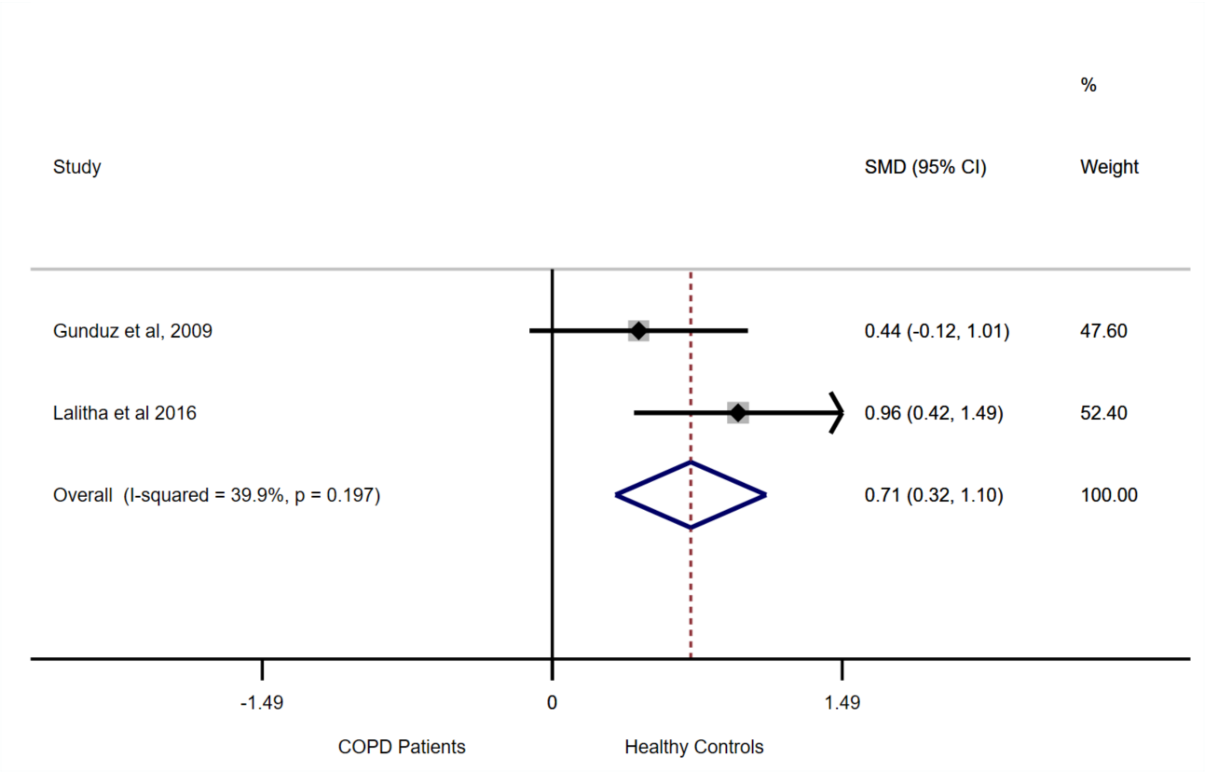

Figure S6. pNN50

Supplemental materials

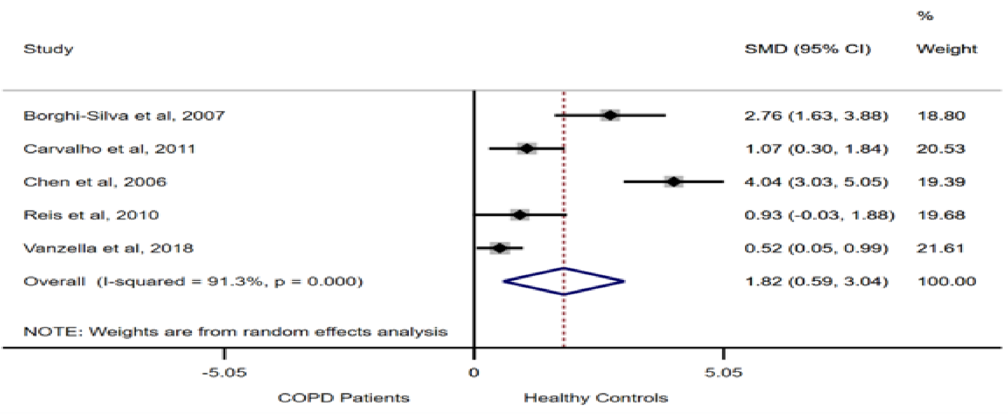

a)

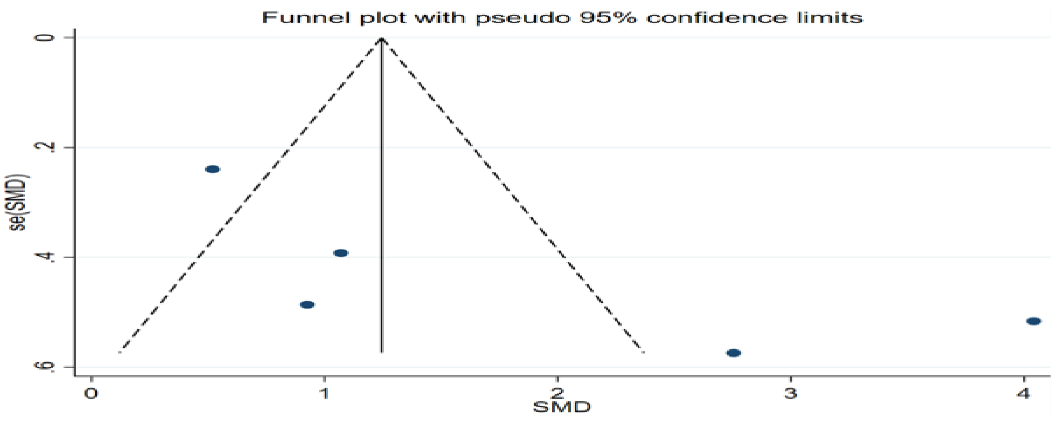

b)

Figure S7. LF forest plot (a) showing the SMD and funnel plot (b) assessing the presence of publication bias.

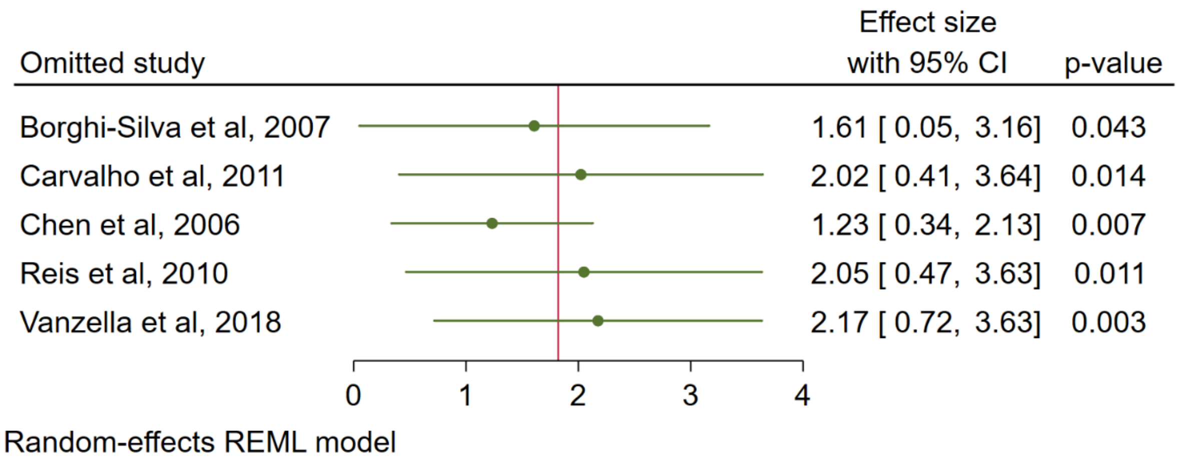

Figure S8. Leave-one-out sensitivity analysis to test whether any of the included studies introduced an exaggerated effect size to the computed LF.

Supplemental materials

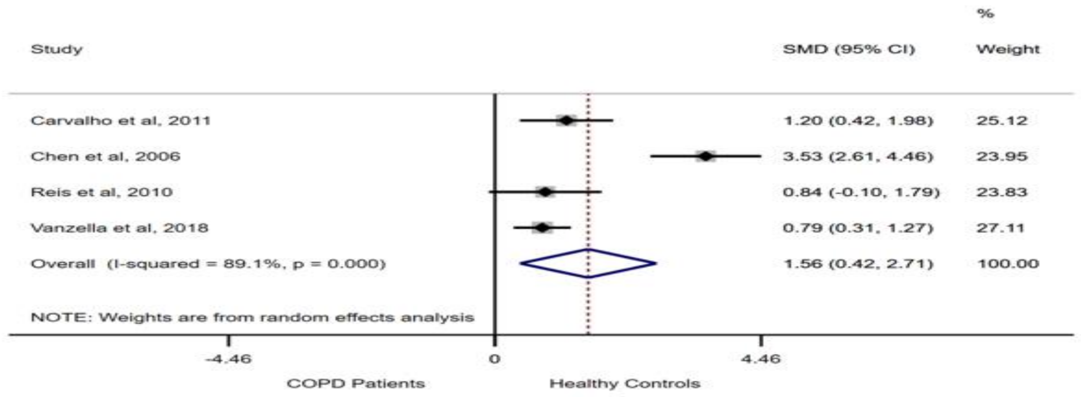

a)

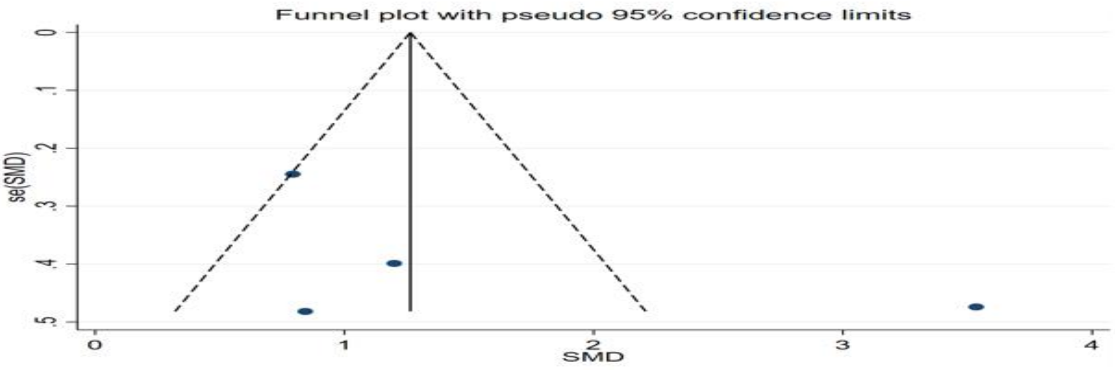

b)

Figure S9. HF forest plot (a) showing the SMD and funnel plot (b) assessing the presence of publication bias.

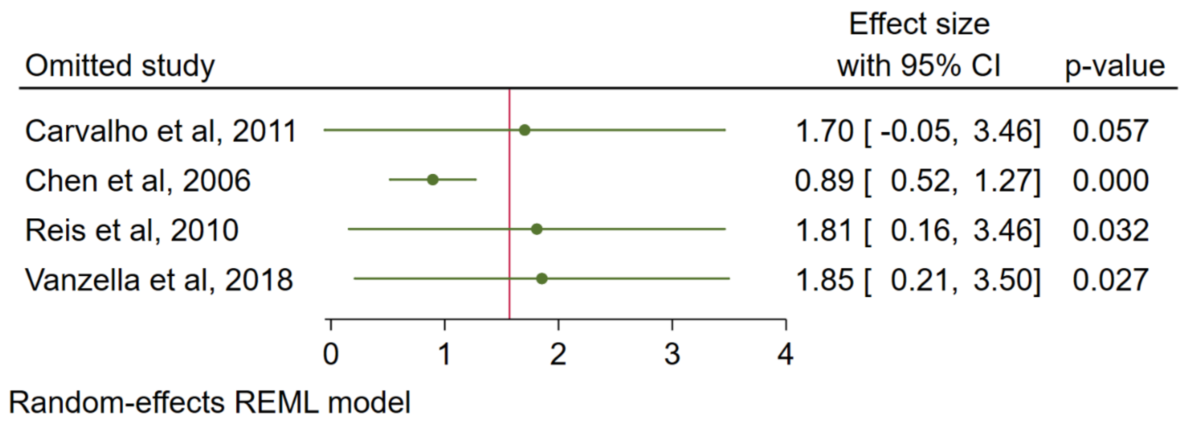

Figure S10. Leave-one-out sensitivity analysis to test whether any of the included studies introduced an exaggerated effect size to the computed HF.

Supplemental materials

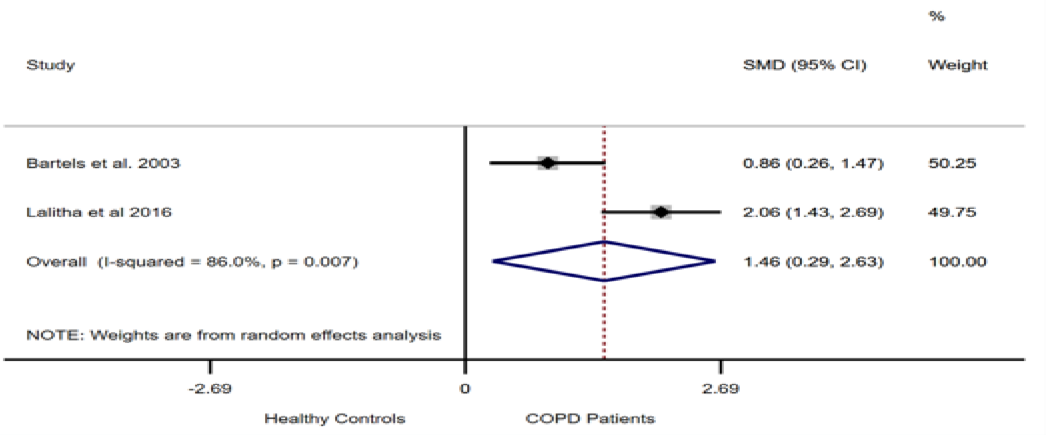

a)

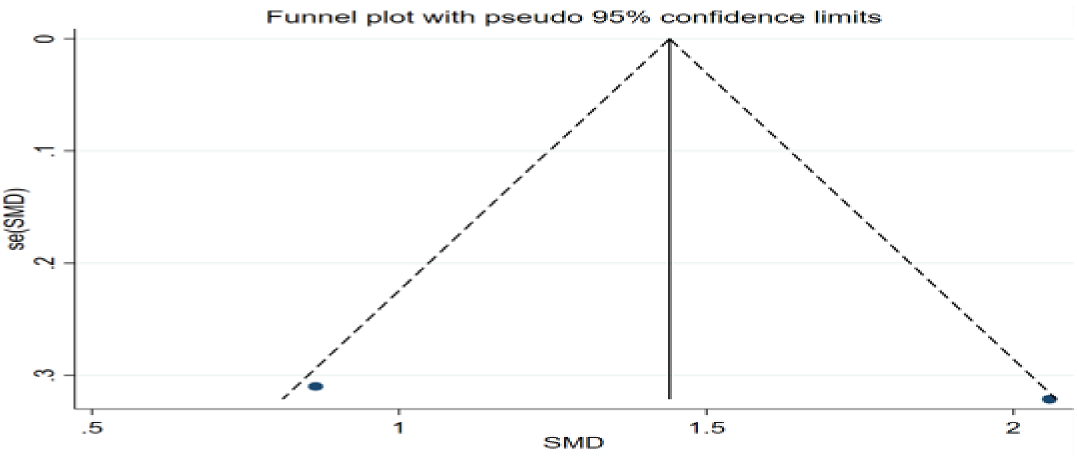

b)

Figure S11. LF: HF Forest plot (a) showing the SMD and funnel plot (b) assessing the presence of publication bias.

Supplemental materials

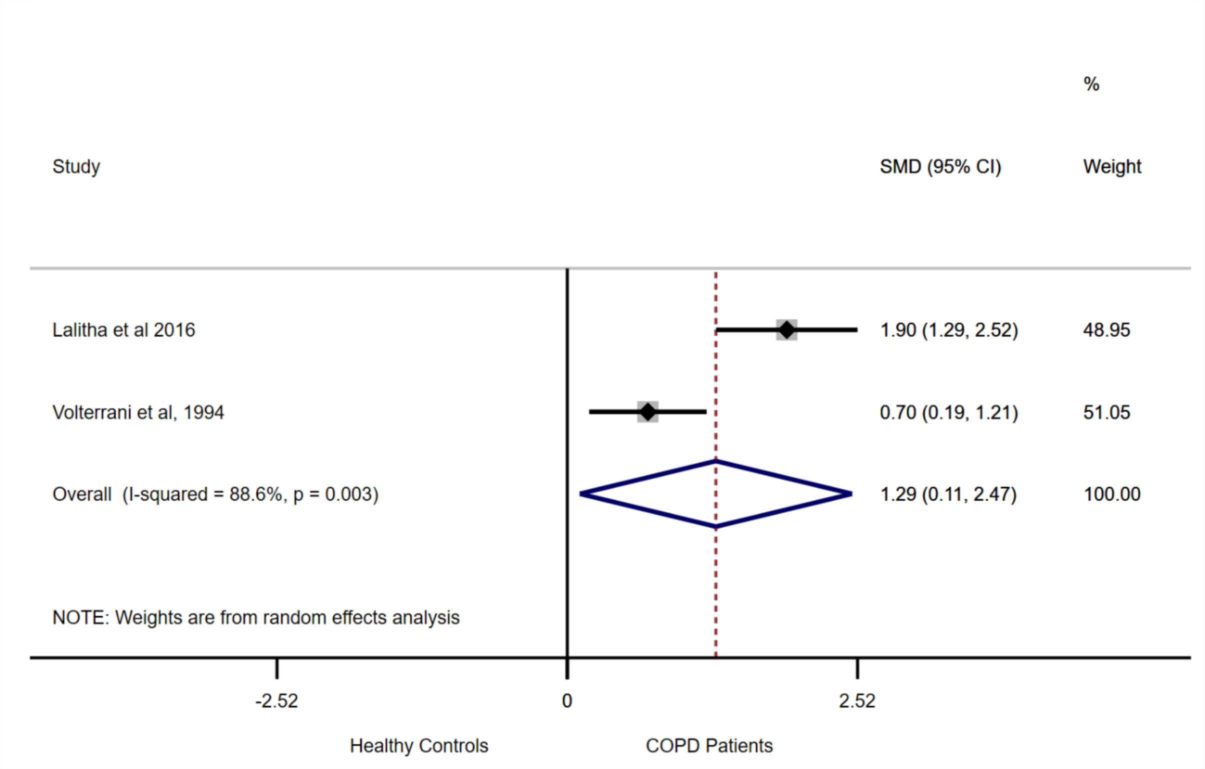

Figure S12. nuHF

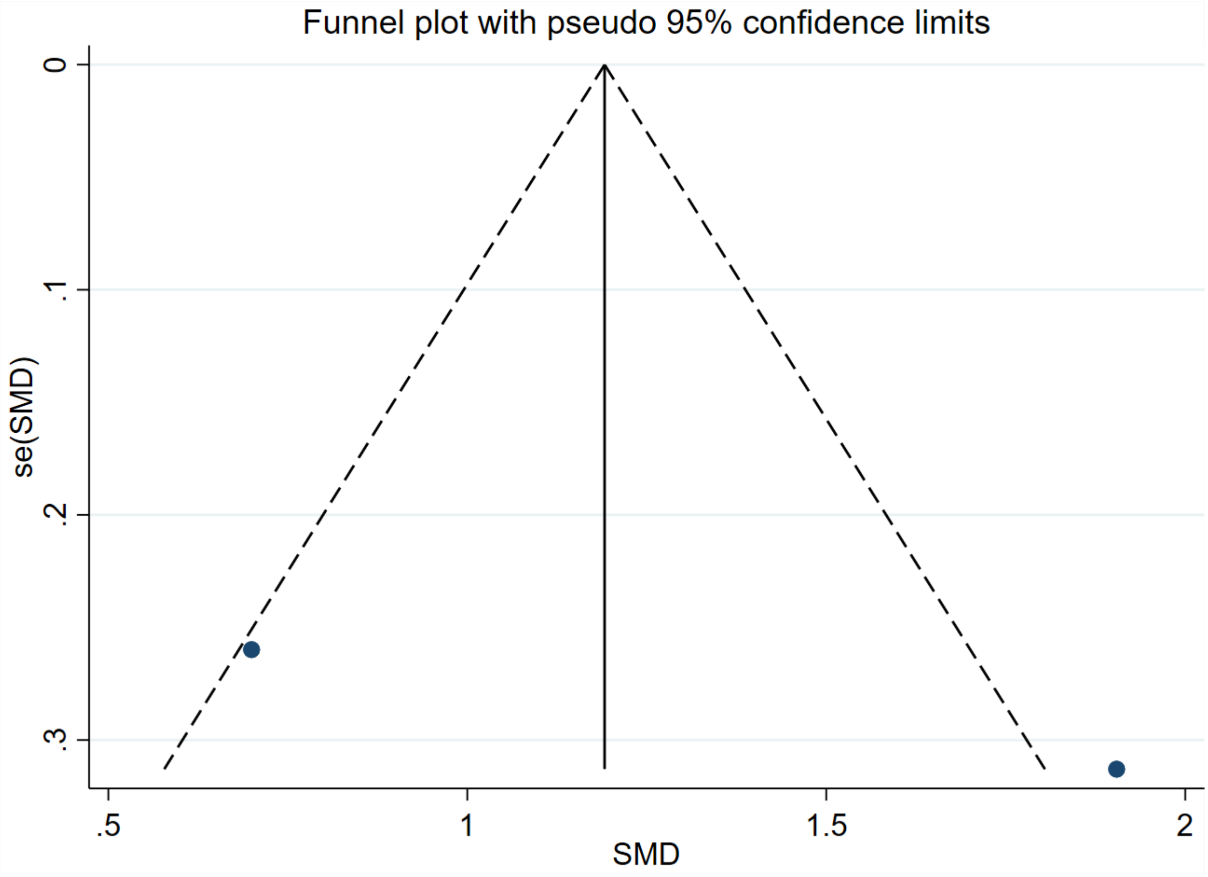

Figure S13. Funnel plot for nuHF.

Supplemental materials

1. Antonelli Incalzi R, Corsonello A, Trojano L, Pedone C, Acanfora D, Spada A, et al. Heart rate variability and drawing impairment in hypoxemic COPD. Brain and Cognition. 2009;70(1):163-70.
2. Bartels MN, Jelic S, Ngai P, Basner RC, DeMeersman RE. High-frequency modulation of heart rate variability during exercise in patients with COPD. Chest. 2003;124(3):863-9.
3. Bedard ME, Marquis K, Poirier P, Provencher S. Reduced heart rate variability in patients with chronic obstructive pulmonary disease independent of anticholinergic or beta-agonist medications. COPD: Journal of Chronic Obstructive Pulmonary Disease. 2010;7(6):391-7.
4. Borghi-Silva A, Reis MS, Mendes RG, Pantoni CBF, Simoes RP, Martins LEB, et al. Noninvasive ventilation acutely modifies heart rate variability in chronic obstructive pulmonary disease patients. Respiratory Medicine. 2008;102(8):1117-23.
5. Camillo CA, Pitta F, Possani HV, Barbosa MVRA, Marques DSO, Cavalheri V, et al. Heart rate variability and disease characteristics in patients with COPD. Lung. 2008;186(6):393-401.
6. Carvalho TD, Pastre CM, de Godoy MF, Fereira C, Pitta FO, de Abreu LC, et al. Fractal correlation property of heart rate variability in chronic obstructive pulmonary disease. International Journal of COPD. 2011;6(1):23-8.
7. Chang ET, Silberstein D, Rambod M, Porszasz J, Casaburi R. Heart rate variability during constant work rate exercise at and above the critical power in patients with severe chronic obstructive pulmonary disease. Tzu Chi Medical Journal. 2011;23(2):42-5.
8. Chen W-L, Chen G-Y, Kuo C-D. Hypoxemia and autonomic nervous dysfunction in patients with chronic obstructive pulmonary disease. Respiratory Medicine. 2006;100(9):1547-53.
9. Corbo GM, Inchingolo R, Sgueglia GA, Lanza G, Valente S. C-reactive protein, lung hyperinflation and heart rate variability in chronic obstructive pulmonary disease - A pilot study. COPD: Journal of Chronic Obstructive Pulmonary Disease. 2013;10(2):200-7.
10. Da Luz Goulart C, Simon JC, De Borba Schneiders P, San Martin EA, Cabiddu R, Borghi-Silva A, et al. Respiratory muscle strength effect on linear and nonlinear heart rate variability parameters in COPD patients. International Journal of COPD. 2016;11(1):1671-7.
11. Gunduz H, Talay F, Arinc H, Ozyildirim S, Akdemir R, Yolcu M, et al. Heart rate variability and heart rate turbulence in patients with chronic obstructive pulmonary disease. Cardiology Journal. 2009;16(6):553-9.
12. Lalitha S, Anandhalakshmi S, Kanimozhi S, Saravanan A. Assessment of heart rate variability in participants with chronic obstructive pulmonary disease. National Journal of Physiology, Pharmacy and Pharmacology. 2017;7(1):85-9.
13. Leite MR, Ramos EMC, Kalva-Filho CA, Rodrigues FMM, Freire APCF, Tacao GY, et al. Correlation between heart rate variability indexes and aerobic physiological variables in patients with COPD. Respirology. 2015;20(2):273-8.
14. Lu WA, Kuo J, Wang YM, Lien TC, Liu YB, Tsai JZ, et al. Reduced enhancement of high-frequency component in the cross spectrum of ECG and nostril airflow signals in patients with chronic obstructive pulmonary disease. Physiological reports. 2016;4(7).
15. Mazzuco A, Medeiros WM, Sperling MPR, de Souza AS, Alencar MCN, Arbex FF, et al. Relationship between linear and nonlinear dynamics of heart rate and impairment of lung function in COPD patients. International Journal of COPD. 2015;10(1):1651-61.
16. Mendes FA, Moreno IL, Durand MT, Pastre CM, Ramos EM, Vanderlei LC. Analysis of cardiovascular system responses to forced vital capacity in COPD. Revista Brasileira de Fisioterapia. 2011;15(2):102-8.
17. Pan L, Wu S, Li H, Xu J, Dong W, Shan J, et al. The short-term effects of indoor size-fractioned particulate matter and black carbon on cardiac autonomic function in COPD patients. Environment International. 2018;112:261-8.
18. Reis MS, Arena R, Deus AP, Simoes RP, Catai AM, Borghi-Silva A. Deep breathing heart rate variability is associated with respiratory muscle weakness in patients with chronic obstructive pulmonary disease. Clinics. 2010;65(4):369-75.

19. Sima CA, Inskip JA, Sheel AW, van Eeden SF, Reid WD, Camp PG. The reliability of short-term measurement of heart rate variability during spontaneous breathing in people with chronic obstructive pulmonary disease. Revista Portuguesa de Pneumologia (English Edition). 2017;23(6):338-42.
20. Stein PK, Nelson P, Rottman JN, Howard D, Ward SM, Kleiger RE, et al. Heart Rate Variability Reflects Severity of COPD in PiZ α1-Antitrypsin Deficiency. Chest. 1998;113(2):327-33.
21. Tseng CY, Chang JCY, Chen YC, Huang HH, Lin CS, How CK, et al. Changes of heart rate variability predicting patients with acute exacerbation of chronic obstructive pulmonary disease requiring hospitalization after Emergency Department treatment. Journal of the Chinese Medical Association. 2018;81(1):47-52.
22. Tukek T, Yildiz P, Atilgan D, Tuzcu V, Eren M, Erk O, et al. Effect of diurnal variability of heart rate on development of arrhythmia in patients with chronic obstructive pulmonary disease. International Journal of Cardiology. 2003;88(2-3):199-206.
23. Van Gestel AJR, Kohler M, Steier J, Teschler S, Russi EW, Teschler H. Cardiac autonomic dysfunction and health-related quality of life in patients with chronic obstructive pulmonary disease. Respirology. 2011;16(6):939-46.
24. Vanzella LM, Bernardo AFB, Carvalho TD, Vanderlei FM, Silva AKFD, Vanderlei LCM. Complexity of autonomic nervous system function in individuals with COPD. Jornal brasileiro de pneumologia : publicacao oficial da Sociedade Brasileira de Pneumologia e Tisilogia. 2018;44(1):24-30.
25. Volterrani M, Scalvini S, Mazzuero G, Lanfranchi P, Colombo R, Clark AL, et al. Decreased heart rate variability in patients with chronic obstructive pulmonary disease. Chest. 1994;106(5):1432-7.
26. Zamarron C, Lado MJ, Teijeiro T, Morete E, Vila XA, Lamas PF. Heart rate variability in patients with severe chronic obstructive pulmonary disease in a home care program. Technol Health Care. 2014;22(1):91-8.
27. Castello-Simões V, Kabbach EZ, Schafauser NS, Camargo PF, Simões RP, Heubel AD, et al. Brain-heart autonomic axis across different clinical status and severity of chronic obstructive pulmonary disease. Respiratory medicine. 2021;185:106511.
